# Hybrid oxide coatings generate stable Cu catalysts for CO_2_ electroreduction

**DOI:** 10.1038/s41563-024-01819-x

**Published:** 2024-02-16

**Authors:** Petru P. Albertini, Mark A. Newton, Min Wang, Ona Segura Lecina, Philippe B. Green, Dragos C. Stoian, Emad Oveisi, Anna Loiudice, Raffaella Buonsanti

**Affiliations:** 1 Laboratory of Nanochemistry for Energy, Institute of Chemical Sciences and Engineering, École Polytechnique Fédérale de Lausanne, Sion, Switzerland; 2 Swiss-Norwegian Beamlines, European Synchrotron Radiation Facility, Grenoble, France; 3 Interdisciplinary Center for Electron Microscopy, École Polytechnique Fédérale de Lausanne, Lausanne, Switzerland

**Keywords:** Colloids, Electrocatalysis, Nanoparticles, Electrocatalysis

## Abstract

Hybrid organic/inorganic materials have contributed to solve important challenges in different areas of science. One of the biggest challenges for a more sustainable society is to have active and stable catalysts that enable the transition from fossil fuel to renewable feedstocks, reduce energy consumption and minimize the environmental footprint. Here we synthesize novel hybrid materials where an amorphous oxide coating with embedded organic ligands surrounds metallic nanocrystals. We demonstrate that the hybrid coating is a powerful means to create electrocatalysts stable against structural reconstruction during the CO_2_ electroreduction. These electrocatalysts consist of copper nanocrystals encapsulated in a hybrid organic/inorganic alumina shell. This shell locks a fraction of the copper surface into a reduction-resistant Cu^2+^ state, which inhibits those redox processes responsible for the structural reconstruction of copper. The electrocatalyst activity is preserved, which would not be possible with a conventional dense alumina coating. Varying the shell thickness and the coating morphology yields fundamental insights into the stabilization mechanism and emphasizes the importance of the Lewis acidity of the shell in relation to the retention of catalyst structure. The synthetic tunability of the chemistry developed herein opens new avenues for the design of stable electrocatalysts and beyond.

## Abstract (web summary)

Active and stable catalysts to accelerate the transition from fossil fuel to renewable feedstocks, reduce energy consumption and minimize environmental footprints are needed. Electrocatalysts based on copper nanocrystals encapsulated in hybrid alumina shells stable against structural reconstruction during CO_2_ electroreduction are reported.

## Main

Hybrid organic/inorganic materials have enabled the discovery of new phenomena and provided solutions to specific needs in many different fields of science^1–3^. Tuning the composition and structure of the organic and inorganic components, along with the interface in between them, modulates the functional properties of the hybrid materials and enables their optimization for the targeted application^1–3^.

The design of active, selective and stable catalysts is one of the biggest challenges to solve in order to build a more sustainable society^4,5^. Currently, a critical need exists to improve the stability of electrocatalysts that enable CO_2_ utilization via the production of chemicals^6–8^.

Copper is one of the most promising catalysts for the generation of products beyond CO (for example, methane, ethylene, ethanol and so on) via the electrochemical CO_2_ reduction reaction (CO_2_RR)^9^. Nanostructuring of copper has improved selectivity with attractive activity compared with bulk^9–12^. However, copper still suffers from drastic and unpredictable reconstruction that often results in performance losses and that masks the inherent sensitivities of the reaction to the geometric structure of the catalyst^13–20^.

The strategies proposed to retain the catalyst structure during CO_2_RR remain scarce^21–23^. A few studies have proposed the incorporation of hetero-elements via alloying^21,22^ or encapsulation using carbon shells^23^, yet these materials have not been made with specific and systematic control over physical structure, which limits the mechanistic understanding.

Generally, fewer solutions to generate stable CO_2_RR electrocatalysts and fundamental studies on catalyst stability exist compared with those in thermal catalysis^24–26^. Studies in thermal catalysis evidence the fundamental importance of fine-tuning the catalyst/support interface and the associated surface chemistry to achieve performant systems while building fundamental knowledge to design stable catalysts^5,27–29^. Such fundamental investigation and knowledge remains decidedly less developed for electrocatalysts.

In this Article, we have prepared copper catalysts encapsulated with alumina (Cu@AlOx) that remain active while being stable against structural reconstruction during CO_2_RR. We developed a colloidal synthetic approach that creates a hybrid organic/inorganic AlOx coating with unique properties. The proposed colloidal synthesis offers an unprecedented structural tunability of this new class of electrocatalysts that enables an in-depth understanding of the mechanism behind the achieved structural stability.

## Synthesis and characterization of the hybrid Cu@AlOx NCs

**Fig. 1:**
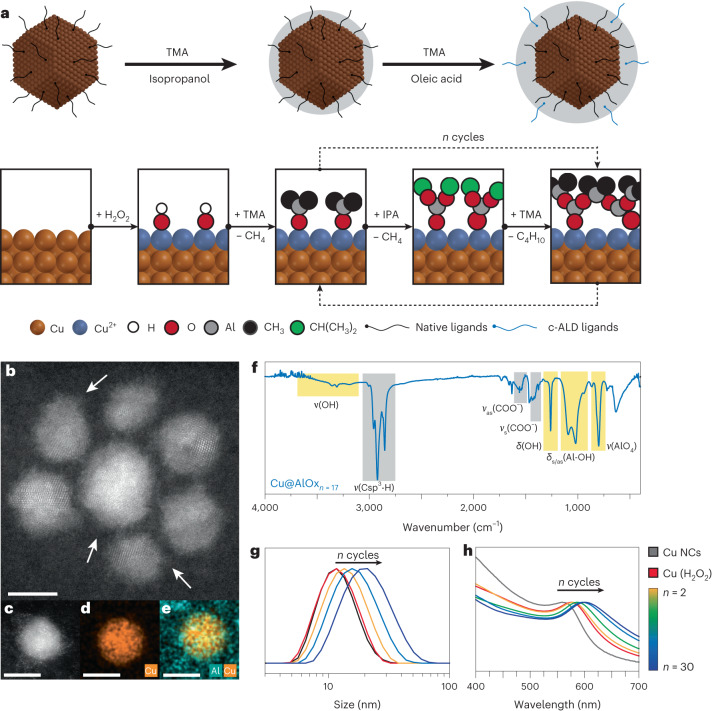
Synthesis and characterization of the hybrid Cu@AlOx NCs. **a**, Schematic of the synthetic protocol. **b**, Representative HAADF-STEM image of Cu@AlOx_*n*=17_ NCs with white arrows pointing at the amorphous alumina coating surrounding the metallic Cu NC core. **c**–**e**, Representative HAADF-STEM image of one particle (**c**) with corresponding EDX elemental map of Cu (**d**) and Cu and Al overlap (**e**); scale bar, 5 nm. **f**, FT-IR spectra of Cu@AlOx_*n*=17_ NCs showing the contribution of the inorganic AlOx shell, highlighted in yellow, and the contribution of the organic ligands (that is TOA, TDPA and OLAC), highlighted in grey. **g**,**h**, Representative DLS and UV–Vis spectra of Cu@AlOx for different *n* cycles (*n* = 2, 8, 17 and 30), which indicates increasing thickness from less than 1 nm for *n* = 2 to 4 nm for *n* = 30 and complete encapsulation for *n* = 17.

In this study, we synthesize electrocatalysts that consist of Cu nanocrystals (NCs) and a hybrid AlOx shell with tunable thickness, which embeds organic ligands. We refer to these materials as Cu@AlOx NCs. We designed a two-step encapsulation process for the synthesis of Cu@AlOx NCs by further developing the colloidal atomic layer deposition (c-ALD) approach (Fig. 1a), which was never applied to oxidation-sensitive metals^30–33^. c-ALD is performed at room temperature under inert conditions in an aprotic solvent. Cu NCs are synthesized via a colloidal chemistry approach and are functionalized with trioctylamine (TOA) and tetradecylphosphonic acid (TDPA) as surface ligands (Supplementary Fig. 1). The first step of the shell growth is a surface treatment with hydrogen peroxide to introduce hydroxyl groups, which anchor the alumina shell on the surface during nucleation (Supplementary Fig. 2). Sequential injections of tri-methyl aluminium (TMA) and isopropanol (IPA) then allow the growth of the alumina shell around the Cu NCs (Supplementary Figs. 3 and 4). The number of ALD cycles (*n*) can be varied to tune the shell thickness. Additional oleic acid (OLAC) ligands are introduced during the growth of the shell to preserve the colloidal stability of the catalysts.

High-angle annular dark-field scanning transmission electron microscopy transmission electron microscopy (HAADF-STEM) and bright-field transmission electron microscopy (BF-TEM) images (Fig. 1b,c and Supplementary Figs. 5 and 6) evidence that the Cu NCs are 7 nm crystalline spheres, each surrounded by an amorphous shell after c-ALD. Energy-dispersive X-ray (EDX) spectroscopy confirms the presence of alumina around the Cu NCs (Fig. 1d,e and Supplementary Fig. 6). Fourier transform infrared (FT-IR) spectroscopy (Fig. 1f and Supplementary Fig. 7) indicates characteristic bands associated to an amorphous/boehmite-like alumina structure^34^. X-ray photoelectron spectroscopy (XPS) corroborates the presence of amorphous alumina by evidencing multiple chemical environments for Al^3+^ (Supplementary Fig. 8 and Supplementary Table 1). In addition to the inorganic component, the FT-IR spectrum shows bands corresponding to the aliphatic organic ligands in shell, which are TOA, TDPA and OLAC. Dynamic light scattering (DLS) confirms the encapsulation of each Cu NC by alumina as well as the shell thickness tunability as a function of the number of cycles, from less than 1 nm to 4 nm from 2 to 30 cycles (Fig. 1g and Supplementary Fig. 9). The local surface plasmon resonance of the Cu NCs redshifts with the number of cycles (Fig. 1h), which results from the change in refractive index caused by the deposition of alumina around Cu NCs. No change is observed after 17 cycles, which indicates a complete encapsulation of the NCs by the shell (Supplementary Note 1).

## Catalytic performance and structural stability during CO_2_RR

Having established the successful outcome of this novel synthetic approach to hybrid metal|oxide NCs, we identified Cu@AlOx_*n*=17_ as the first candidate for the catalytic studies as full encapsulation of the Cu NCs promises better structural stability. As-synthesized 7 nm spherical Cu NCs were used as a reference catalyst. These NCs represent an ideal platform to interrogate and assess the impact of the AlOx shell on catalytic performance. Indeed, while not being state-of-the-art catalysts in terms of selectivity towards the desired C_2+_ products, the 7 nm Cu spheres undergo drastic and rapid reconstruction that is accompanied by changes in the CO_2_RR product distribution^11,16–19^.

**Fig. 2:**
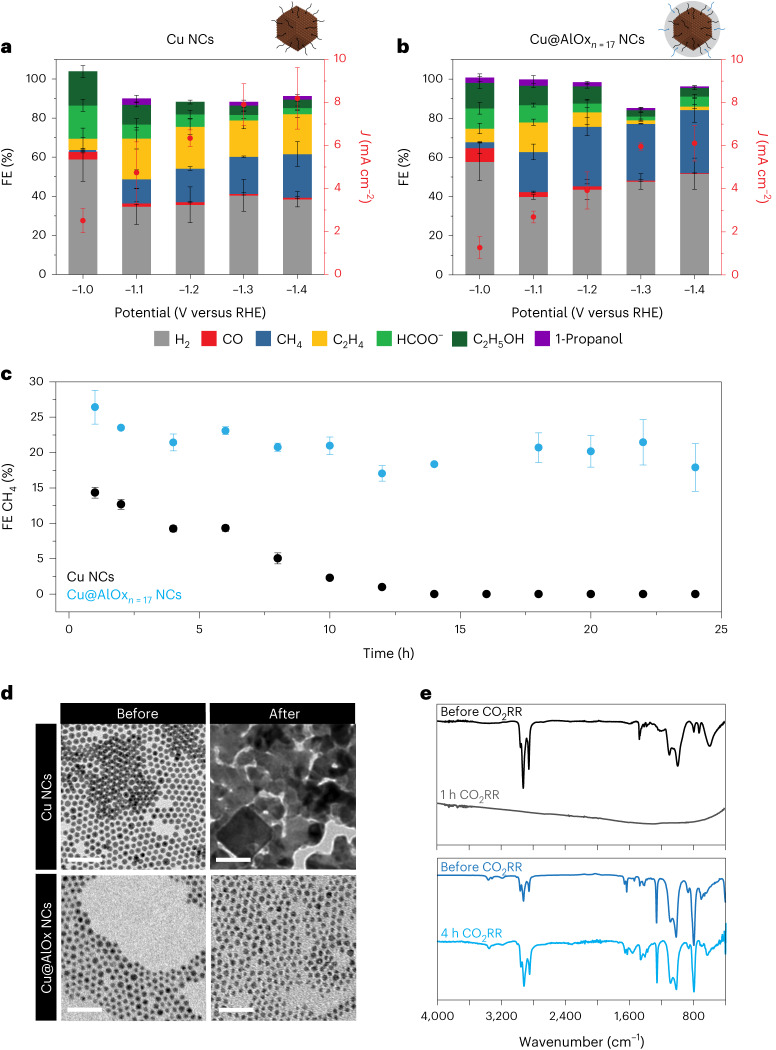
CO_2_RR performance and post-CO_2_RR characterization of Cu NCs and Cu@AlOx NCs. **a**,**b**, Total FEs for all gaseous products (that is, H_2_, CO, CH_4_ and C_2_H_4_) and the main liquid products (that is, HCOO^−^, C_2_H_5_OH and 1-propanol) of the as-synthesized Cu NCs (**a**) and of the Cu@AlOx_*n*=17_ NCs (**b**). The data are the average of three independent experiments, and the error bars are the calculated standard deviation. **c**, Temporal evolution of the FE for methane for as-synthesized Cu NCs (black) and Cu@AlOx_*n*=17_ NCs (blue) at −1.1 V versus RHE. The data are the average of six measurements (every 2 h), and the error bars are the calculated standard deviation. **d**, Representative BF-TEM image of Cu NCs and Cu@AlOx_*n*=17_ NCs before (left) and after 4 h CO_2_RR at −1.1 V versus RHE (right), respectively. Scale bars, 50 nm. The as-synthesized Cu NCs transform into aggregated structures, with copper oxide cubes forming upon air exposure, consistently with previous literature^16–19^. **e**, FT-IR spectra of Cu NCs and Cu@AlOx_*n*=17_ post-4 h of chronoamperometry at −1.1 V versus RHE.

Interestingly, the Cu@AlOx_*n*=17_ NCs exhibit a total current density that is in the same order of magnitude of the as-synthesized Cu NCs (Fig. 2a,b, Supplementary Figs. 10–12 and Supplementary Table 2). This result highlights the unicity of the c-ALD grown alumina because a dense coating of ceramic alumina would be otherwise an insulator and prevent any electron transfer, which means zero current. As for the selectivity, the Cu NCs generate preferentially ethylene over methane, which is consistent with previous studies^11,16–19^. On the contrary, the Cu@AlOx_*n*=17_ NCs promote methane over ethylene across the entire potential range. The methane selectivity of the Cu@AlOx_*n*=17_ NCs remains stable for at least 24 h, while a decay to zero occurs for the Cu NCs over 10 h (Fig. 2c and Supplementary Fig. 13). The rapid loss of the Cu NCs is line with the dynamic reconstruction of copper, which is detrimental for methane active sites.

The most striking result comes from the post-CO_2_RR TEM analysis (Fig. 2f and Supplementary Fig. 14). The as-synthesized Cu NCs undergo full reconstruction into scrambled catalysts during the start-up phase of electrolysis as the potential ramps to −1.1 V versus reversible hydrogen electrode (RHE), which is consistent with previous studies^16–19^. On the contrary, the Cu@AlOx_*n*=17_ NCs retain their initial morphology with irrelevant changes in particle size distribution (Supplementary Fig. 14).

In a complementary manner, post-CO_2_RR FT-IR analysis (Fig. 2e,f) confirms these results as the ligand characteristic peaks disappear for the Cu NCs, which is consistent with previous work^35^, while the characteristic structural bands of the alumina shell and those of the organic ligands remain intact for the Cu@AlOx_*n*=17_ NCs, which is also shown by linear sweep voltammetry (Supplementary Fig. 15). Similarly, post-CO_2_RR XPS corroborates the presence of AlOx and of nitrogen-containing ligands (Supplementary Fig. 16 and Supplementary Table 3).

## Mechanism behind the structural stability during CO_2_RR

The observation that Cu@AlOx NCs are active for CO_2_RR, with selectivity for methane, and are stable against structural reconstruction, which is not the case for the as-synthesized Cu NCs, prompted us to interrogate the reason behind this unique behaviour.

**Fig. 3:**
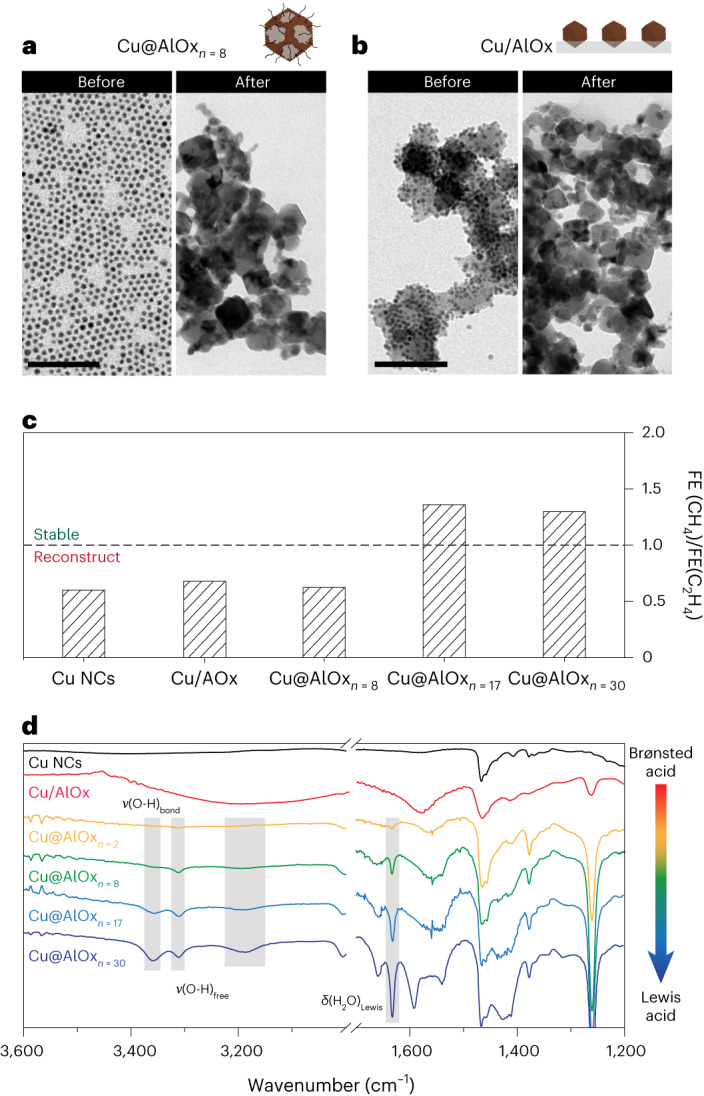
Effect of the catalyst morphology on the reconstruction behaviour. **a**,**b**, Representative BF-TEM image of Cu@AlOx_*n*=8_ (**a**) and Cu/AlOx (**b**) before (left) and after 1 h CO_2_RR at −1.1 V versus RHE (right), respectively. Scale bars, 100 nm. **c**, Ratio FE(CH_4_)/FE(C_2_H_4_) as function of the catalyst morphology and c-ALD cycles. **d**, FT-IR spectra of Cu@AlOx_*n*=x_ for *n* = 2, 8, 17 and 30 and Cu/AlOx, with *ν*(OH, free) at 3,360, 3,316 and 3,192 cm^−1^ and *δ*(H_2_O) Lewis at 1,630 cm^−1^ highlighted in grey. The peaks in the 1,550–1,450 cm^−1^ region correspond to OLAC added during the shell growth.

First of all, we aimed at addressing the importance of the coating morphology, including shell thickness and spatial distribution of the Cu and alumina components. Thus, we exploited the versatility of the c-ALD to synthesize Cu@AlOx_*n*=8_ and Cu/AlOx, which are Cu NCs with partial encapsulation and Cu NCs supported on alumina, respectively (Supplementary Fig. 17). Neither one of the samples preserved their initial morphology nor exhibited preferential methane selectivity (Fig. 3a–c and Supplementary Fig. 18). By contrast, Cu@AlOx_*n*=30_ NCs did not reconstruct and favoured methane production similarly to Cu@AlOx_*n*=17_ (Fig. 3c and Supplementary Fig. 18). These results indicate that a minimum number of c-ALD cycles are needed to prevent catalyst reconstruction during CO_2_RR and to induce preferential methane selectivity. They also highlight that simply using alumina as a support does not work in the same way of the encapsulation.

We gained additional insight into the chemical nature of the AlOx shell by collecting FT-IR spectra on samples synthesized with different c-ALD cycles (Fig. 3d and Supplementary Fig. 19). The data reveal a critical change from a broad bell-shape signal at around 3,200 cm^−1^ into three peaks at 3,360, 3,316 and 3,192 cm^−1^. This change indicates a transition from hydrogen-bonded (Brønsted acidity) to scarcer repartition (free hydroxyl, Lewis acidity) as the alumina shell grows, which mimics a recent study in gas-phased ALD^36^. The Lewis acid character of the thicker shells is further confirmed by the increased intensity of the bending mode of H_2_O (*δ*(HOH)) at 1,630 cm^−1^, which is characteristic of residual water Lewis coordinated to surface cations^34^. The changes accompanying the transition from Brønsted to Lewis acidity are even more pronounced when comparing Cu/AlOx to Cu@AlOx wherein a change from AlO_6_ to AlO_4_ coordination environments is observed, the latter being identified as a Lewis acidic site (Supplementary Fig. 19)^37^.

Second, we performed operando X-ray absorption spectroscopy (XAS) for the as-synthesized Cu NCs, the Cu@AlOx_*n*=17_ and the Cu/AlOx, the latter is a reference example where the Cu|AlOx interface is unable to prevent reconstruction of copper (Fig. 4 and Supplementary Figs. 20–27).

**Fig. 4:**
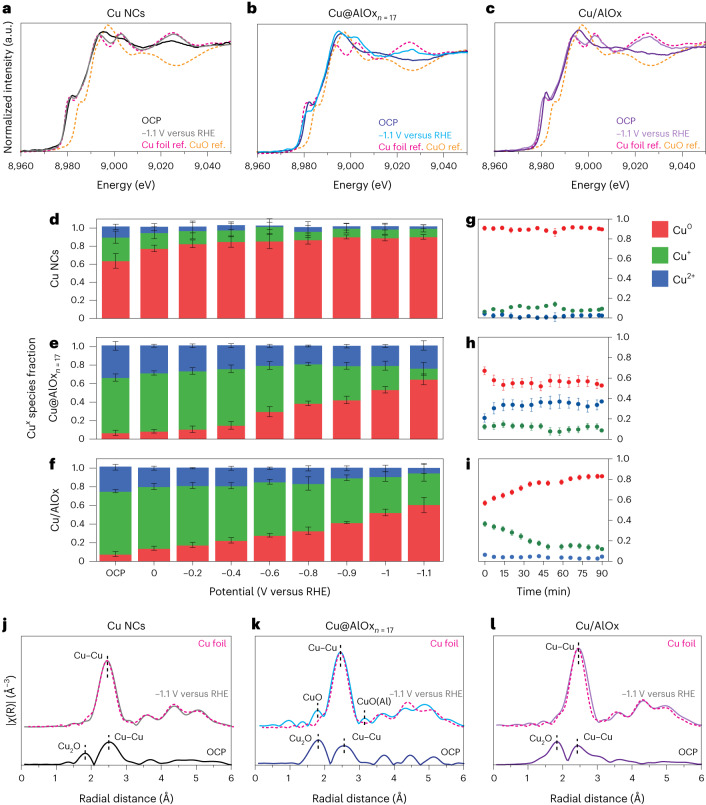
Structural characterization of the Cu|AlOx interface by operando XAS. **a**–**c**, Cu K-edge XANES spectra at OCP (dark colour) and at −1.1 V versus RHE (bright colour) for Cu NCs (**a**, black), Cu@AlOx_*n*=17_ (**b**, blue) and Cu/AlOx (**c**, purple), respectively, together with Cu foil (pink dashed line) and CuO (orange dashed line) standard. **d**–**i**, Evolution of Cu^*x*^ species fraction as a function of the applied potential (**d**–**f**) and time at −1.1 V versus RHE (**g**–**i**) extracted from linear combination analysis of the XANES spectra for Cu NCs. The data are the average of copper fractions extracted from six consecutive XANES spectra, and the error bars are the calculated standard deviation: Cu NCs (**d** and **g**), Cu@AlOx_*n*=17_ (**e** and **h**) and Cu/AlOx (**f** and **i**). **j**–**l**, EXAFS data at at OCP (dark colour) and at −1.1 V versus RHE (bright colour) for Cu NCs (**j**, black), Cu@AlOx_*n*=17_ (**k**, blue) and Cu/AlOx (**l**, green).

The X-ray absorption near-edge structure (XANES) profiles of the Cu K-edge indicate a mixture a copper species (Cu^0^, Cu^+^ and Cu^2+^) at open circuit potential (OCP) for all samples (Fig. 4a–c). Substantial differences emerge in the behaviour of the samples during CO_2_RR. Indeed, the XANES spectra collected at −1.1 V versus RHE exhibit the characteristic white line and pre-edge features of metallic copper for Cu NCs and for Cu/AlOx while the white line of Cu@AlOx_*n*=17_ suggests the retention of copper oxide with no evidence of bulk CuAl_2_O_4_ formation (Supplementary Figs. 20 and 21).

The evolution of the copper speciation with the applied potential (Fig. 4d–f and Supplementary Fig. 22) provide additional insight. First of all, the fraction of oxidized copper at OCP is similar in Cu@AlOx_*n*=17_ and Cu/AlOx cases, yet this fraction is much higher compared with that measured for the as-synthesized Cu NCs. As the cathodic potential is applied, the level of oxidized copper is reduced for all samples; however, Cu@AlOx_*n*=17_ and Cu/AlOx maintain a substantial fraction of Cu^2+^ and Cu^+^, respectively. Cu@AlOx_*n*=17_ preserves most of the Cu^2+^ during operation over time; on the contrary, Cu/AlOx eventually transforms into metallic Cu with a statistically insignificant fraction of oxidized copper remaining (Fig. 4g–i and Supplementary Fig. 23). Cu@AlOx_*n*=8_ behaves similarly to Cu/AlOx, which is that the copper eventually evolves into fully metallic (Cu^0^) state during CO_2_RR (Supplementary Figs. 24 and 25).

Operando extended X-ray absorption fine structure (EXAFS) confirmed the trends observed in the XANES (Fig. 4j–l, Supplementary Figs. 26 and 27 and Supplementary Table 4). At OCP, all samples exhibit clear Cu–O scattering peaks, which can mostly be attributed to Cu_2_O, along with those attributed to Cu–Cu from the metallic face-centered cubic (FCC) Cu. The Cu–O peak is more intense for the two samples containing alumina, which is consistent with the higher fraction of Cu^+^ in the XANES. During CO_2_RR, only the Cu@AlOx_*n*=17_ NCs show scattering peaks corresponding to Cu–O. The peak at 1.9 Å matches well with CuO, which is in line with the XANES. Instead, the peak at 3.1 Å is a unique non-FCC feature that cannot be fully ascribed to any bulk standard. This feature (indicated as Cu–O(Al) in Fig. 4k) is positioned between CuO and CuAl_2_O_4_; as such, it probably reflects the interaction between oxidized copper (Cu^2+^) and the alumina network. These peaks are present in both Cu@AlOx_*n*=17_ and Cu/AlOx at OCP, yet convoluted underneath the dominant Cu_2_O scattering. However, they disappear under CO_2_RR for both samples.

Overall, a clear correlation between the stabilization of Cu^2+^ under cathodic potentials and the morphological stabilization emerges in the Cu@AlOx_*n*=17_ NCs.

Current knowledge identifies redox processes and the resulting dissolution/reprecipitation of soluble transient Cu intermediate species as the main drivers for the copper reconstruction during the start-up and shut-down of the electrochemical cell and during CO_2_RR, respectively^15,19,38^. The Cu@AlOx_*n*=17_ locks the oxidation state of a significant portion of the copper surface, which, as a result, becomes less prone to the redox processes accounting for the Cu reconstruction (Supplementary Figs. 28 and 29). Furthermore, the interface between Cu and AlOx greatly enhances the surface adhesion energy of the surface copper atoms^39^; a surface copper more strongly bound to the bulk will be less prone to intermediate-induced dissolution during CO_2_RR.

However, if metal–support interactions and the formation of Cu–O–Al bonds were the only cause behind the structural stability, Cu@AlOx_*n*=8_ and Cu/AlOx should have shown some improvement compared with the Cu NCs, which is not what we observe. The complete shell acting as a mechanical barrier against copper atom mobility is unlikely considering the amorphous and porous nature of the c-ALD deposited alumina shell (Supplementary Fig. 30). Indeed, physical shielding is generally claimed as the explanation to prevent reconstruction of electrocatalysts covered by thick and dense coatings^40–42^. Thus, we must resort to the structural and chemical differences between Cu@AlOx_*n*=8_, Cu/AlOx and Cu@AlOx_*n*=17_ to explain their different behaviour_._

The FT-IR data suggest that the alumina evolves from Brønsted to Lewis character as a function of the number of c-ALD cycles. The Cu/AlOx sample possesses a Bronsted character similar to that of the thinner shells, including Cu@AlOx_*n*=8_. Generally, a more pronounced Lewis character renders the alumina more resistant to dealumination, which occurs under the basic pH that is expected near the surface of the catalyst^6,37^. The transition from Cu^2+^ to Cu^+^ and eventually to Cu^0^ with cathodic potential and time observed in the XANES data of the Cu/AlOx during CO_2_RR is consistent with such a dealumination process. Thus, a correlation emerges between the Lewis acidity of the alumina in the Cu@AlOx_*n*≥17_, along with the chemical stability of the alumina itself deriving from it, and the locking of the Cu^2+^ and structural stability of the catalysts under CO_2_RR.

Furthermore, we speculate that the ligands embedded in the shell also play a key role in the structural stability of the Cu@AlOx NCs by conferring the porosity and mechanical flexibility, which are crucial to mass transfer and withstand gas generation at the interface between solid catalyst, liquid electrolyte and gas reagent and products.

## Intrinsic activity and manipulation of selectivity

Cu|oxide materials are emerging as catalysts with overall promising performance in CO_2_RR in terms of steering selectivity and achieved catalyst stability (Supplementary Note 2)^43–47^. However, the fundamental insight into their behaviour has been limited so far because of the large variability and/or poorly defined and not systematically tuned compositional and structural features in the catalytic materials utilized in most of these studies. The well-defined and characterized Cu@AlOx NCs provide the opportunity to go beyond the current state of the art and build a more rational framework around this class of CO_2_RR catalysts.

The identification of Cu^2+^–O–Al with AlO_4_ coordination (Lewis acid) as a key motif for the structural stabilization indicates that catalysis takes place on neighbouring copper sites.

**Fig. 5:**
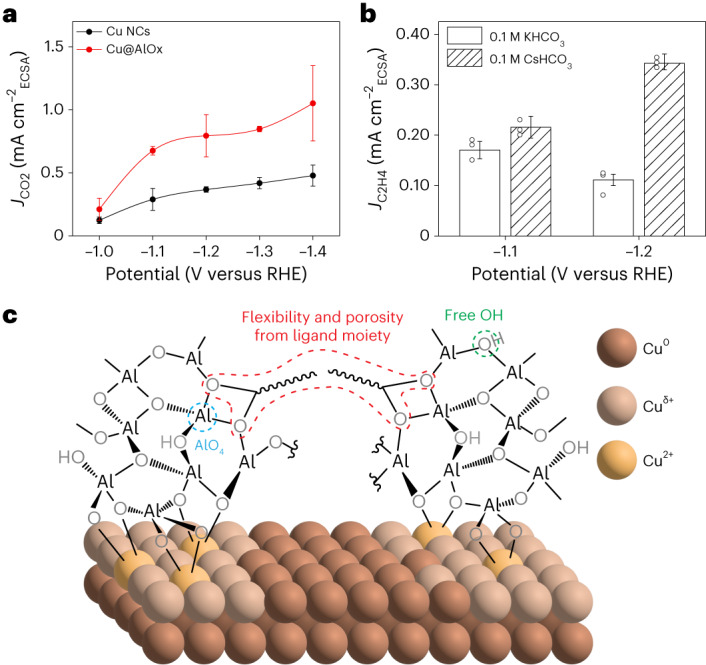
Intrinsic activity and manipulation of selectivity for Cu|oxide interfaces. **a**, Partial current density of CO_2_ for Cu NCs and Cu@AlOx_*n*=17_ normalized by ECSA. **b**, Partial current density of C_2_H_4_ for Cu@AlOx_*n*=17_ in 0.1 M KHCO_3_ and 0.1 M CsHCO_3_ normalized by ECSA. The data are the average of three independent experiments (individual data points are reported in the graph) and the error bars are the calculated standard deviation. **c**, Schematic representation of the hybrid organic/inorganic AlOx coating with the key chemical and structural features.

The current density normalized by the electrochemically active surface area (*J*_ECSA_) indicates that the intrinsic activity of these sites doubles compared with that of the Cu NCs (Fig. 5a and Supplementary Figs. 31 and 32). The methane intrinsic activity is boosted five times, while the hydrogen remains similar compared with Cu NCs (Supplementary Fig. 32). We speculate that Cu sites with depleted electronic density in closer proximity to the Cu^2+^–O–Al generate methane^48^. These sites must coexist with fully metallic Cu that continues to produce hydrogen.

Data on partial current densities indicate that C–C coupling is not intrinsically suppressed (Supplementary Fig. 32). The latter observation confirms that electronic effects explain the enhanced activity for methane rather than C–C coupling being hindered by spatial confinement. In addition to changes in the oxide to tune electronic effects, microenvironment manipulation emerges as a future strategy to modulate and steer the selectivity of the Cu@AlOx catalysts towards products different than methane. In fact, changing the electrolyte from KHCO_3_ to CsHCO_3_ evidences increased ethylene production from the Cu@AlOx NCs (Fig. 5b and Supplementary Fig. 33). This result provides an additional evidence that the productivity towards C_2+_ products can be enhanced.

Thus, an overall atomistic picture of the Cu|AlOx interface emerges along with the structure of the active sites (Fig. 5c). These same motifs might be extendable to other Cu|metal oxide catalysts wherein the less defined character of the material previously studied prevented those to be identified (Supplementary Note 2)^43–47^. Thus, catalyst modifications that increase the fraction of these structural motifs can eventually lead to overall enhanced catalytic productivity for ethylene in future studies.

As the alumina coating chemistry on the copper surface is foreseen as generalizable to other oxides, organics ligands and metal surfaces, the novel material platform introduced in this work holds the potential to generate stable and active catalysts with tunable selectivity for CO_2_RR by framing general guideline. Indeed, changing the Lewis acidity of the metal oxide coating and microenvironment is expected to steer selectivity^47^. Intentional manipulation of the surface oxidation state of the metal core could be exploit to expand the catalysts for CO_2_RR beyond copper^49^. The integration of molecular wires as the organic component might provide the opportunity to moderate the intrinsic lower electronic conductivity of the shell^50^. Ultimately, the same synthetic strategy could be applied to create other hybrid materials in a more tunable and defined way for applications beyond catalysis.

## Method

### Synthesis of Cu NCs and H_2_O_2_ treatment

Cu NCs were synthesized following a previously reported protocol^16^. The as-synthesized Cu NCs were oxidized by introducing a dilute solution of hydrogen peroxide in ethanol (in 1:1 ratio H_2_O_2_ to Cu atoms on the surface). The mixture was allowed to react for 20 min before purifying the reaction mixture by centrifugation at 20,980*g* for 8 min and redispersion in octane.

### Synthesis of Cu@AlOx NCs

Cu(H_2_O_2_) NCs were diluted in 9 ml of octane to reach a concentration of 0.6 mmol l^−1^ of copper and stirred under N_2_ flow. One c-ALD cycle consists of the following: (1) dropwise addition of TMA diluted in hexane to the NC suspension (the optimized rate of the syringe pump was fixed at 1 ml h^−1^); (2) 5 min waiting time; (3) dropwise addition of IPA; (4) 5 min waiting time. The TMA concentrations range from 80 μmol l^−1^ to 0.4 mmol l^−1^ depending on the growth stage. Stock solutions of IPA and OLAC diluted in octane were prepared with concentrations of 0.8 mmol l^−1^ and 2.5 mmol l^−1^, respectively.

### Synthesis of Cu/AlOx

The synthesis of Cu/AlOx was performed similarly to as Cu@AlOx but with a 3.3 mmol l^−1^ TMA solution. Upon the first cycles the colloidal stability is rapidly loss to form a NCs supported on alumina.

### Electrochemical measurements

The catalysts were drop-casted to achieved a mass loading of 15 µg on 1.33 cm^2^ glassy carbon. Electrochemical measurements were performed using a custom-made H-cell described in previous study^35^ using 0.1 mol l^−1^ KHCO_3_, a flow rate of 5 ml min^−1^ with glassy carbon as working electrode, Ag/AgCl as reference electrode and platinum foil as counter electrode where both compartment were separated by a (Selemion AMV) membrane. Note that H_2_ from the competing hydrogen evolution reaction was observed as the main product with a faradaic efficiency (FE) exceeding 75% for both Cu NCs and Cu@AlOx NCs below −1 V versus RHE. Gas products (H_2_, CO, CH_4_ and C_2_H_4_) were quantified using gas chromatography, and liquid products were quantified using high-performance liquid chromatography. Additional information on the electrochemical measurements can be found in Supplementary Information.

Ultraviolet–visible (UV–Vis) absorption measurements were performed in transmission mode using a PerkinElmer Lambda 950 spectrophotometer equipped with a deuterium lamp as a light source for the ultraviolet range and a tungsten halide lamp as a light source for the visible and infrared ranges, and a photomultiplier tube with a Peltier-controlled InGaS detector. Samples were measured in screw-top, gas-tight quartz cuvettes (path 10 mm) and were prepared by diluting in 2 ml octane a Cu NC stock suspension. Background spectra were recorded for 2 ml clean octane solvent.

DLS measurements were carried out using a Zetasizer Nano ZS (Malvern) instrument. The Nano ZS system is equipped with a 4 mW red laser (633 nm) and a detection angle of 173°. The samples were prepared in a 2 ml glass cuvette in 1.2 ml octane solvent. For each sample, three measurements were performed with auto-optimization from the software. All measurements are reported in intensity.

FT-IR spectroscopy was carried out on a PerkinElmer Two spectrometer using an attenuated total reflectance plate. Air was used as a background spectrum. Samples were prepared by drop-casting octane suspensions of the Cu NCs directly onto the attenuated total reflectance plate and leaving to air-dry. Spectra were recorded with a resolution of 0.5 cm^−1^ and a total of 16 scans.

Bright-field TEM images were recorded on a JEOL-2100F using a beam energy of 120 keV. Samples were drop-casted on a copper TEM grid (Ted Pella) before imaging.

HAADF-STEM images and EDX spectrum images were acquired on a Thermo Fischer Scientific Tecnai-Osiris and a double Cs-corrected Titan-Themis transmission electron microscopes operated in scanning mode at an accelerating voltage of 200 kV. These microscopes are equipped with a high-brightness X-FEG, Super-X EDX acquisition system comprised of four silicon drift detectors and TIA/Bruker-Esprit and Velox acquisition software, respectively.

XAS experiments were performed at the Swiss-Norwegian beamlines BM31 at the European Synchrotron Radiation Facility in France. The catalyst suspension was drop-casted onto a thin (2.5 × 2.5 × 0.5 mm^3^) glassy carbon support and a Kapton window allowed the X-rays to pass through. For standards and other ex situ measurements (typically using pressed pellets with the sample diluted in a light matrix such as boron nitride or cellulose to obtain an appropriate thickness), XAS was collected in transmission using ionization chambers for transmission detection. The measurements were carried out in fluorescence mode at an incident angle of approximately 45°. A Si(111) double crystal monochromator was used to condition the beam from the bending magnet source. Fluorescence X-ray absorption near edge structure (XANES) spectra were acquired using a Vortex single-element silicon drift detector with XIA-Mercury digital electronics and a time resolution of 1 min per spectrum. The resulting XAS data were reduced and normalized using the Prestopronto package or PAXAS. Subsequent analysis of the extracted EXAFS data was performed using EXCURV (v. 9.3).

## Online content

Any methods, additional references, Nature Portfolio reporting summaries, source data, extended data, supplementary information, acknowledgements, peer review information; details of author contributions and competing interests; and statements of data and code availability are available at 10.5281/zenodo.10524037.

### Supplementary information

Supplementary Information Supplementary Figs. 1–33, Tables 1–4 and Notes 1 and 2.

## Data Availability

Experimental data are openly available in Zenodo at 10.5281/zenodo.10524037.
