# Supplementary material for: Hybrid oxide coatings generate stable Cu catalysts for CO2 electroreduction
Source: Nat Mater. 2024 Feb 16;23(5):680–7. doi: 10.1038/s41563-024-01819-x (PMC11068572; doi:10.1038/s41563-024-01819-x)
Supplement: Supplementary file 1 — Supplementary Figs. 1–33, Tables 1–4 and Notes 1 and 2. [file 41563_2024_1819_MOESM1_ESM.pdf]

nature materials

Article

https://doi.org/10.1038/s41563-024-01819-x

Hybrid oxide coatings generate stable Cu catalysts for CO_2 electroreduction

In the format provided by the authors and unedited

Table of contents

Synthetic Methods 4

Additional Instrumentation 7

Figure S1: Surface characterization of as-synthesized 7 nm spherical Cu NCs 10

Figure S2: Surface characterization of Cu(H_2O_2) 12

Figure S3: Change in LSPR of c-ALD grown AlOx using O_2 versus IPA 14

Figure S4: Optimization of c-ALD first cycles 15

Figure S5: Bright Field TEM images of Cu@AlOx$_{n=17}$ 17

Figure S6: HAADF-STEM images together with EDX elemental map of Cu@AlOx 18

Figure S7: Full-range FT-IR spectra 20

Figure S8: XPS Spectra of Cu 3s/Al 2s region 22

Figure S9: DLS characterization 24

Figure S10: Comparison of the CO_2RR performance of Cu NCs and of Cu@AlOx$_{n=17}$ 28

Figure S11: Overall CO2RR performances of Cu(H_2O_2) NCs 29

Figure S12: Representative TEM images of Cu(H_2O_2) NCs after 1 hour of CO_2RR 30

Figure S13: Long Chronoamperometry test 32

Figure S14: Representative TEM images of Cu and Cu@AlOx$_{n=17}$ NCs after CO_2RR 33

Figure S15: Post 4 hours CO_2RR XPS spectra 34

Figure S16: LSV of the as-synthesized Cu NCs and Cu@AlOx$_{n=17}$ NCs. 35

Figure S17: Representative HAADF-STEM image of Cu/AlOx 37

Figure S18: Evaluation upon partial encapsulation and metal/support Cu|AlOx NCs 38

Figure S19: FT-IR spectra of the Cu/AlOx compared with Cu@AlOx 39

Figure S20: Cu K-edge XANES spectra of commercial references 40

Figure S21: LCA on XANES spectra 41

Figure S22: Evolution of operando XANES as function of the applied potentials 43

Figure S23: Evolution of operando XANES as function of time at -1.1 V vs RHE 45

Figure S24: Ex-situ XAS data of Cu@AlOx$_{n=8}$ 46

Figure S25: Evolution of Cu^{+2} fraction from OCP to -1.1 V vs RHE 47

Figure S26: k^3-weighted EXAFS spectra at OCP (dark color) and at -1.1 V vs RHE 48

Figure S27: Phase-corrected FT(k^3)-EXAFS spectra 49

Figure S28: SWV of Cu NCs vs Cu@AlOx$_{n=17}$ NCs 51

Figure S29: Cyclic voltammograms of Cu, Cu@AlOx, Cu/AlOx NCs and $CuAl_2O_4$ 53

Figure S30: FT-IR spectra of Cu and Cu@AlOx with pyridine 55
Figure S31: Evaluation of the ECSA for Cu and Cu@$AlOx_{n=17}$ NCs 57
Figure S32: Partial current density for Cu and Cu@$AlOx_{n=17}$ NCs 59
Figure S33: Comparison of the CO2RR performance of Cu@AlOxn=17 NCs in 0.1M KHCO3 and 0.1M CsHCO3 60

Supplementary Note 1 25
Supplementary Note 2 56

Table S1: Detailed XPS peak analysis 23
Table S2: FEs of as-synthesized Cu NCs, Cu(H_2O_2) and Cu@$AlOx_{n=17}$ NCs 31
Table S3: Detailed XPS peak analysis 36
Table S4: Structural data extracted from analysis of Cu K-edge EXAFS 50

References 61

Synthetic Methods

Materials

Copper(I) acetate (Cu(OAc), 98%), tri-n-octylamine (TOA, technical grade, 98%), oleic acid (OLAC, technical grade, 90%), tetradecylphosphonic acid (TDPA, 97%), octane (OT, anhydrous, ≥99%), toluene-d8 (Tol-d8, 99.5 atom % D), isopropanol (IPA, anhydrous, 99.5%) ethanol (EtOH, anhydrous, 95%), hydrogen peroxide (H_2O_2, 30 % (w/w) in H_2O, contains stabilizer) were purchased from Sigma Aldrich. Hexane (anhydrous, >96%) was purchased from TCI Deutschland GmbH and trimethylaluminum (TMA, 98%) was purchased from Strem. Potassium carbonate (K_2CO_3, 99+ %, ACS reagent) was obtained from Acros organics. OLAC was degassed by stirring under dynamic at 130°C during a 1-hour vacuum before storing in an N_2-filled glove box.

Methods

General consideration:

All syntheses and manipulations of Cu NCs were performed under a dry N_2 atmosphere, using Schlenk-line techniques or a glove box. Anhydrous organic solvents were used for the manipulation, analysis and storage of Cu NCs. All volumes below 20mL were measured and dispensed using Eppendorf microliter pipettes. All glassware were stored in oven at 110ºC to minimize air/moisture contamination.

Synthesis of copper nanocrystals (Cu NCs):

Cu NCs were synthesized following a protocol adapted from Huang et al.[1] In a typical synthesis, tri-n-octylamine (20 mL) was introduced in a 50mL three-necked round bottom flask and then degassed under dynamic vacuum at 130ºC for 1 hour while stirring. The flask was then refilled with N_2 gas and cooled to 50ºC. Tetradecyl-phosphonic acid (270 mg, 1 mmol) and copper(I) acetate (245 mg, 2 mmol) were added to the flask as a powder, forming a green, cloudy mixture. The mixture was heated to 180°C and held for 30 minutes, during which the mixture turned light brown. After 30 minutes, the mixture was rapidly heated to 270°C, quickly forming a dark red mixture. After 30 minutes at 270°C, the dark red colloidal suspension was allowed to cool down to room temperature by removing the heating mantle. Then, the reaction mixture was transferred into 40 mL glass vials with a septum filled with N_2 via a 20 mL plastic syringe. To each 5 mL portion of the crude reaction mixture, hexane (5 mL) and ethanol (15 mL) were added. The particles were isolated by centrifugation at

13500 rpm for 8 minutes, and the supernatant was discarded. The particles were then washed using hexane (5 mL) and ethanol (15 mL) and again isolated after centrifugation. Finally, the particles were redispersed and combined into a single suspension using 10 mL octane and were stored in an N_2-filled glovebox. From ICP-OES a typical concentration was around 10-15 mg/mL of Cu.

Surface treatment with hydrogen peroxide ($Cu(H_2O_2)$ NCs):

50 µL of H_2O_2 (30 % (w/w) in H_2O (10 mol.L^{-1}) were introduced in a 20 mL glass. The glass vial was closed with a septum cap and flushed with N_2 for 5 min. In the glovebox, EtOH (10mL, anhydrous) was added to the vial to obtain a solution of 0.05 mol.L^{-1} of H_2O_2. A diluted suspension of Cu NCs (6.75 mg of Cu NCs, 0.75 µmol.L^{-1} of NCs) in octane (9mL, anhydrous) was prepared in a second 20 mL glass vial equipped with a stirrer bar. While stirring the as-prepared Cu NCs suspension, 313 µL of H_2O_2 were added. The reaction mixture was stirred for 15 min in the glovebox. The particles were then transferred in a 50 mL centrifuge tube with EtOH (20 mL) isolated by centrifugation at 13500 rpm for 8 min. Finally, the particles were redispersed in 1-2 mL of octane and stored in the glovebox. The $Cu(H_2O_2)$ NCs were observed to remain colloidally stable for months with a stable position of the plasmon resonance peak in the UV-Vis spectrum.

Colloidal Atomic Layer Deposition:

The following steps were performed in the glovebox:

An initial stock solution of trimethyl-aluminum (TMA) was prepared in a 4 mL glass vial by diluting pure TMA (200 µL, 5.2 mol.L^{-1}) in octane (600 µL, anhydrous) to reach a concentration of 2.6 mol.L^{-1} of TMA. Successive dilutions were performed to obtain 3.3 mmol.L^{-1}, 0.4 mmol.L^{-1} and 80 µmol.L^{-1}solutions of TMA.

A 9 mL suspension of $Cu(H_2O_2)$ NCs was prepared in a 20 mL glass vial with a stir bar and a septum cap, by diluting $Cu(H_2O_2)$ NCs stock suspension (340µg) in octane to reach a concentration of 0.6 mmol.L^{-1} of copper.

Stock solutions of isopropanol (IPA) and oleic acid (OLAC) diluted in octane were prepared with concentrations of 0.8 mmol.L^{-1} and 2.5 mmol.L^{-1} respectively.

The following steps were performed outside the glove box with vials connected to N_2 line from the Schlenk line:

A gas-tight glass syringe (S.G.E. Gas Tight Luer Lock Syringe 5mL) with a stainless steel 304 syringe from Sigma Aldrich was first charged with 2 mL of 2.6 $mol.L^{-1}$ TMA to scavenge residual impurities. The syringe was charged at 0.5 $mL.min^{-1}$ using a syringe pump. The syringe was discharged manually to remove residual air bubbles. The syringe was then rinsed with 2 mL of 80 $\mu mol.L^{-1}$ solutions of TMA (0.5 $mL.min^{-1}$) and still discharged manually. The syringe was finally refilled with 2 mL of 80 $\mu mol.L^{-1}$ solutions of TMA.

The general protocol of c-ALD proceeds as follows. The suspension of diluted $Cu(H_2O_2)$ NCs was stirred gently under N_2. The first cycle consists of drop-wise (1 $mL.hr^{-1}$) addition of 200 µL of 80 $\mu mol.L^{-1}$solution of TMA. The injection of TMA is followed by a 5 min delay to allow complete reaction before injecting 10 µL of IPA 0.8 $mmol.L^{-1}$ diluted with 90µL of octane with a 1 mL plastic syringe. The reaction is left to proceed for 5 minutes before starting the new addition of 200 µL of 80 $\mu mol.L^{-1}$solution of TMA. Once the TMA injection is completed and after 5 min waiting time, 10 µL of 2.5 $mmol.L^{-1}$ OLAC diluted with an additional 90 µL of octane is injected.. At the end of the two cycles, the obtained suspension ($Cu@AlOx_{n=2}$) should remain stable with no aggregates suspended or attached to the stir bar. If not, the volume of injected TMA needs to be optimized, specifically it should be decreased in steps of 25 µL in each test. The following cycles consist of alternating injections of 150uL of 0.4 $mmol.L^{-1}$ TMA and 80 µL of 0.8 $mmol.L^{-1}$ IPA diluted with 80µL of octane. Every three cycles, the IPA is replaced by 50 µL of 2.5 $mmol.L^{-1}$ OLAC diluted with an additional 50 µL of octane. The shell thickness can be tuned by the number of cycles. The growth per cycle can be increased by injecting a bigger volume of TMA (e.g. 0.4 $mmol.L^{-1}$).

The exact same steps were undertaken for Cu/AlOx with the difference of performing the c-ALD with 3.3 $mmol.L^{-1}$ TMA for every injection. 5-6 cycles were usually performed to ensure that all the spheres were supported on alumina.

Additional Instrumentation

Nuclear magnetic resonance spectroscopy (NMR). Solution NMR measurements were recorded on a Bruker Avance III HD 400 MHz 9.4 T spectrometer equipped with a BBFO liquid probe. One-dimensional (1D) 1H spectra were acquired using a standard pulse sequence from the Bruker library.

X-ray photoelectron spectra (XPS) were recorded using an Axis Supra (Kratos Analytical) instrument, using the monochromated Kα X-ray line of an Al anode. The pass energy was set to 20 eV with a step size of 0.1 eV. The samples were electrically insulated from the sample holder and charges were compensated. Spectra were referenced at 284.8 eV using the C–C bound of the C 1s orbital. Cu NCs samples were prepared by drop-casting nanocrystal films onto clean Si substrates.

Electrochemical setup:

Electrochemical measurements were performed using a Biologic SP-300 potentiostat. Ambient pressure CO_2 electrolysis was carried out in a custom-made gas-tight electrochemical cell made of polycarbonate and fitted with Buna-N O-rings. The configuration of the electrochemical cell was such that the working electrode sat parallel with respect to the counter electrode to ensure a uniform potential distribution across the surface. The geometric surface area for both of the electrodes was 1.33 cm^2. A Selemion AMV anion exchange membrane was used to separate the anodic and cathodic compartments. Each of the compartments in this cell contained a small volume of electrolyte (2 mL each) to concentrate liquid products and therefore increase detection limits. 0.1 $mol.L^{-1}$ $KHCO_3$ solution was used as electrolyte. To make such a solution, a 0.05 $mol.L^{-1}$ K_2CO_3 solution was bubbled for 30 min with CO_2. Before CO_2 electrolysis was conducted, the electrolyte in the cathodic compartment was purged with CO_2 for at least 30 min. During electrolysis, CO_2 was constantly flowed through the electrolyte at a flow rate of 5 sccm to prevent depletion of CO_2 in the electrolyte and to allow continuous analysis of gaseous products via a gas chromatograph. The flow rate of CO_2 was controlled with a mass flow controller (Bronkhorst), and the gas was first humidified with water by passing it through a bubbler to minimize evaporation of electrolyte. Platinum foil was used as the counter electrode and Ag/AgCl electrode (leak free series) from Innovative Instruments, Inc. was used as the reference. Voltages were converted to the RHE scale by using a calibrated reference electrode (equation 1).

$$E(\text{V vs RHE}) = E(\text{ref}) + 0.206 + 0.0591 \times pH - (R_{\text{cell}} \times I)$$

where E(ref) is the recorded potential against the Ag/AgCl reference electrode (V); +0.206 V is the Ag/AgCl reference electrode correction; R_{cell} is the ohmic resistance between the working and the reference electrode, which was defined using electrochemical impedance spectroscopy (EIS) analysis prior to the measurement (Ohm); I is the imposed current (A). Details on the EIS experiments can be found in our previous study.[2]

Cyclic voltammetry was carried out using the same H-cell set-up, sweeping the potential in the cathodic direction from −1 V to + 0.5 V vs Ag wire, at a scan rate of 25 mV s^{-1}. Detail in the Square Wave Voltammetry can be found in our previous study.[2]

For all values, error bars, representing standard deviations obtained with triplicate measurements, are given.

Product Quantification

A gas chromatograph (GC, SRI Instruments) equipped with a HayeSep D porous polymer column, thermal conductivity detector, and flame ionization detector was used for the analysis of gaseous products. The GC was calibrated for H_2, CO, CH_4, C_2H_4, and C_2H_6. Five standard gas mixtures (Carbagas) were used to obtain the calibration plots for gaseous-product concentration determination. Ultra-high purity N_2 (99.999%) was used as a carrier gas.

The Faradaic efficiency (FE) for the gaseous products is calculated with the following equation

$$\text{FE}=n\text{e}\times F\times C\times f\times PR\times T\times I$$

where ne is the number of electrons transferred to the product formation, F is the Faraday constant (96485 $C.mol^{-1}$), C is the measured concentration of the product by GC (in ppm), f is the gas flow rate ($mL.s^{-1}$), P is the pressure (1.013×10^5 Pa), I is the imposed current (in A), R is the universal gas constant (8.314 $J.mol^{-1}.K^{-1}$), and T is the temperature (K). Values obtained after 10 min of electrolysis are not included in the calculation of Faradaic efficiency as the GC gas flow is not yet stabilized at that point. The length of the electrolysis is set for 3900 seconds to allow an average of seven injections.

The Faradaic efficiency of liquid products was determined in a similar manner. A volume of 0.25 mL of both the catholyte and the anolyte was collected and analyzed with high-

performance liquid chromatography (HPLC) on an UltiMate 3000 instrument from Thermo Scientific, which is equipped with a refractive index detector for product quantification and Aminex HPX-87H (BioRad) column for product separation (1 mM H_2SO_4 was used as the eluent). The necessity to collect electrolyte from both compartments is reasoned by the possible product crossover from the catholyte to the anolyte (i.e., formic acid and acetic acid, which exist in their anionic forms, namely, formate and acetate, in the neutral solution and can migrate through the anion exchange membrane, which separates the two compartments).

The resulting formula used for calculations of the liquid products' FE is

$$\mathrm{FE}=n\mathrm{e}\times F\times CV\times I\times t$$

where ne is the number of electrons transferred to the product formation, F is the Faraday constant, C is the measured concentration of the product by HPLC (mol.L^{-1}), V is the cell volume (4 mL), I is the measured current (A), and t is the duration of electrolysis (3900 s).

For all values, error bars representing standard deviations obtained with triplicate measurements are given.

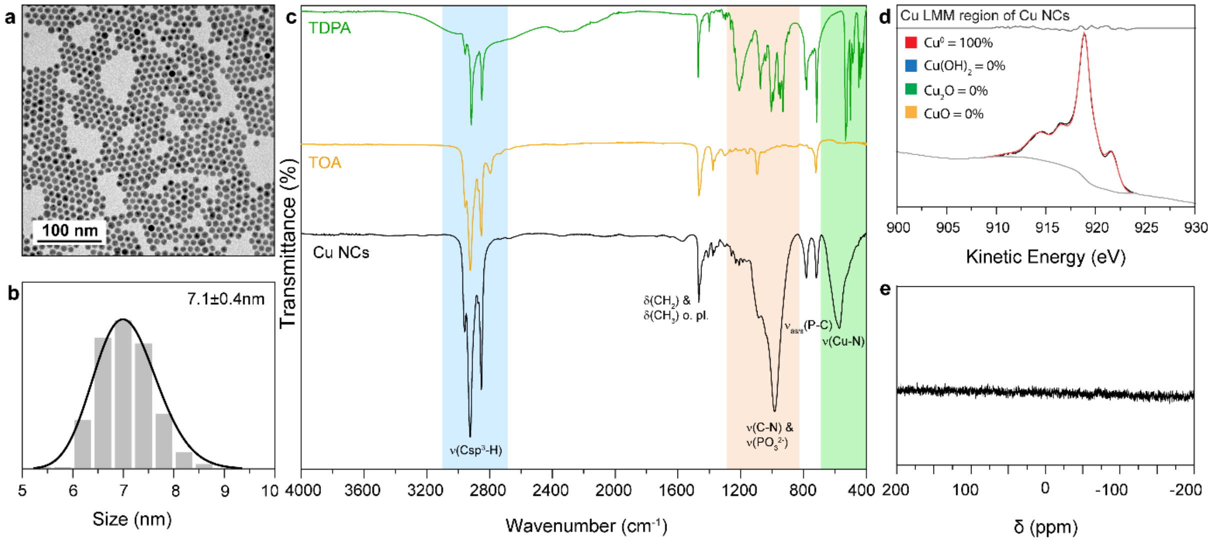

Figure S1. Surface characterization of as-synthesized 7 nm spherical Cu NCs. a, Representative BF-TEM of as-synthesized Cu NCs together with **b,** their size distribution bar graph, obtained by measuring >500 nanocrystals in the BF-TEM images using Fiji software for automatic sizing and fitting lognormal distribution. **c,** FT-IR spectrum of the same sample together with TOA and TDPA spectrum. The blue, orange and green regions highlight the ν(Csp^3-H), ν(PO_3^{2-}), ν(C-N) and ν(Cu-N) broad signals, respectively. **d,** Auger spectrum of Cu LMM region of Cu NCs. **e,** $^{31}P\{^1H\}$ NMR spectra of Cu NCs, measured in toluene-d_8.

Figure S1a,b show a representative BF-TEM image with the corresponding size distribution of Cu NCs, which is 7.1±0.4 nm. As explained by Loiudice *et al.* and Segura Lecina *et al.*, the native surface chemistry of the NCs plays a pivotal role in the growth of metal-oxide shells by c-ALD .[3–5]

Fourier transform infrared spectroscopy (FT-IR), and X-ray photoelectron spectroscopy (XPS) were performed to decipher the nature of the capping agent and the oxidation state of copper on the surface of the Cu NCs. **Figure S1c** reports the FT-IR spectrum of the Cu NCs. The region between 3000 – 2750 cm^{-1}, highlighted in blue, corresponds to the antisymmetric and symmetric stretching mode of the carbon hybridized Csp^3 (ν(Csp^3-H), CH_3, s-CH_2 and as-CH_2) from the alkyl chains contained in both, TOA and TDPA.[6] The band at 1468 cm^{-1} is characteristic of the CH_2 bending mode (δ(CH_2)) and the CH_3 out of plane bending mode, (δ(CH_3) o. ph.) again, contained in both ligands.[7] The broad and intense band at 985 cm^{-1} with a shoulder at 1092 cm^{-1} highlighted in orange can be respectively attributed to the PO_3^{2-} stretching modes (ν(PO_3^{2-})), indicative of tridentate coordinated of TDPA on the NCs

surface, and the shoulder to the C-N stretching mode (ν(C-N)) from TOA.[7,8] Additionally, the two bands at 785 and 720 cm^{-1} are attributed to the asymmetric and symmetric P-C stretching mode (ν(P-C)).[8] Finally, the broad and intense band at 580 cm^{-1} is indicative of the stretching mode of the copper-nitrogen bond (ν(Cu-N)), from bounded TOA to the Cu NCs surface.[9,10] From the FT-IR spectrum, we conclude that the as-synthesized Cu NCs are capped by both TOA and TDPA. However, a quantification of the two ligands is not possible by FT-IR as the peak intensities depend not only on the amount but also on the strength of the dipolar moment of the bond. Understanding the proportion of ligands and the type of bonding between them and the surface is of particular importance as one of them (i.e. TDPA) can potentially act as anchoring points for the nucleation of the shell, while the other (i.e. TOA) cannot.

XPS indicates that Cu NCs have a metallic surface with no detected trace of Cu^{+} or Cu^{2+} as shown by the Cu LMM region with a characteristic multiplicity of metallic copper in **Figure S1d**.[11,12] Accordingly, TDPA is most likely electrostatically bound on the surface, rather than covalently, and/or is only present in minor quantity. Indeed, some oxidation of Cu would be detected to the presence of $Cu^{+/+2}$ – O – P bonds if TDPA was covalently bound. Additionally, no signal was detected in the ^{31}P NMR of Cu NCs (**Figure S1e**), which indicates that TDPA is not present in significant amount and with labile bonds on the surface of the Cu NCs. Therefore, TDPA cannot fulfil the role of anchoring point for the nucleation of the shell. Furthermore, no nucleation of TMA can occur directly on copper surfaces[13] and no reaction is foreseen between TOA and TMA. As a matter of fact, attempts to perform c-ALD on as-synthesized Cu NCs were unsuccessful.

Having learned that, it became clear that the surface chemistry of the as-synthesized Cu NCs needed to be modified to enable the nucleation and growth of alumina. An oxygen-containing group had to be introduced on the surface to provide anchoring points for the organometallic precursor used during c-ALD. In that sense, Gharachorlou *et al.* have demonstrated that an oxide layer was necessary to grow alumina on copper foil by gas-phase ALD.[13] The growth of metal-oxide shells on metallic NCs by sol-gel approaches has also been possible only after tuning the surface of the NCs to interact with the metal-oxide precursors by introducing hydroxyl groups on the surface.[14,15] Thus, a surface treatment with H_2O_2 was performed on the as-synthesized Cu NCs.

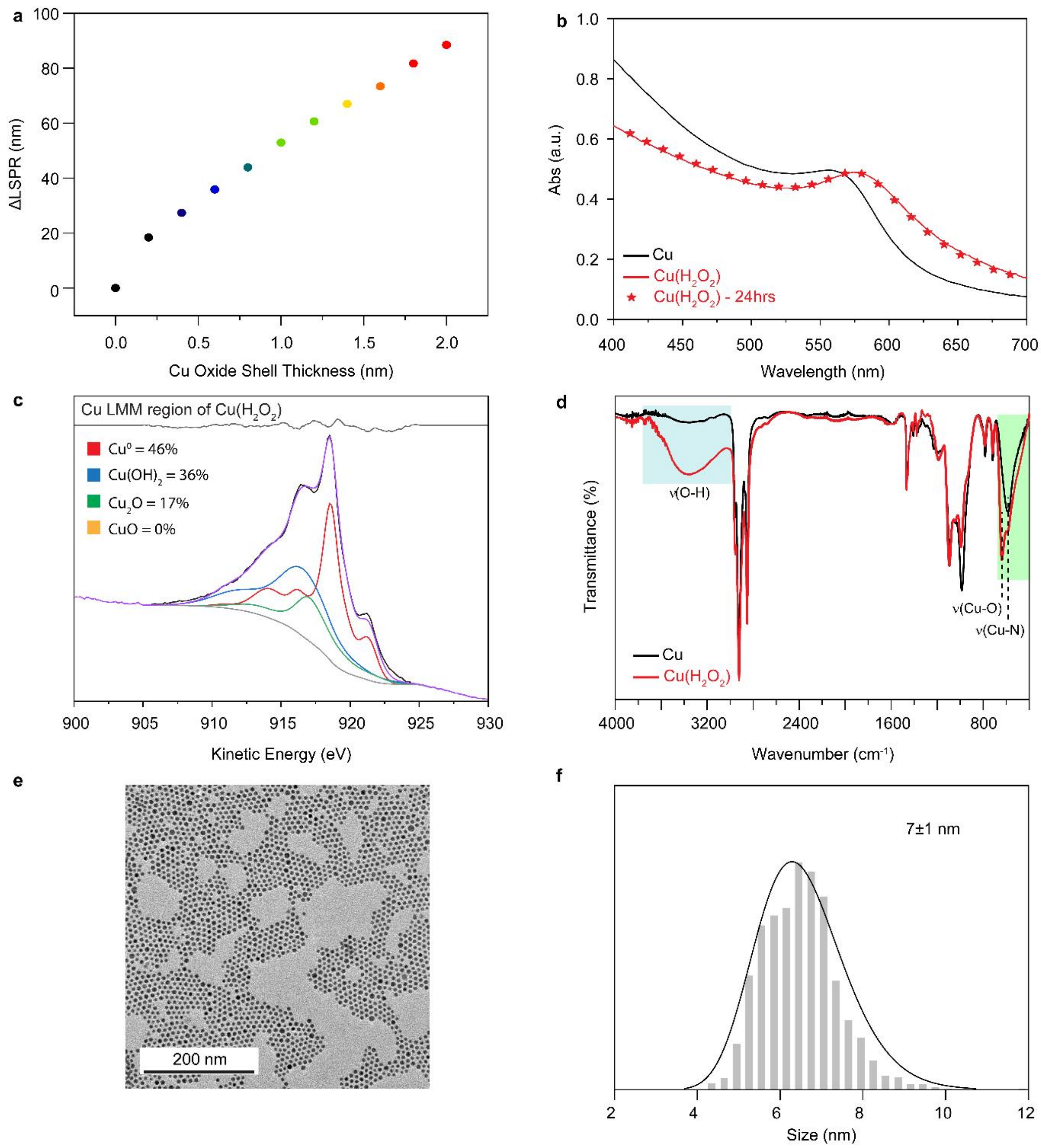

Figure S2. Surface characterization of $Cu(H_2O_2)$ a, Simulation of the evolution of LSPR peak as a function of the CuO shell thickness. **b,** UV-VIS spectra of Cu NCs and $Cu(H_2O_2)$. **c,** XPS spectra of Cu LMM region together with the fit. **d,** FT-IR spectra. The blue and green regions highlight the ν(O-H) and ν(Cu-O) and ν(Cu-N) broad signals, respectively. **e,** and **f,** TEM images of $Cu(H_2O_2)$ (**e**) with corresponding size distribution (**f**) obtained by measuring >500 nanocrystals in the TEM images using Fiji software and fitting lognormal distribution.

Assuming 7 nm Cu NCs and a refractive index of of copper oxide of 2.6 (CuO and Cu2O having similar refractive index), simulations were conducted to correlate the shift of the

localized surface plasmon resonance (LSPR) peak of Cu NCs with the thickness of the copper oxide shell formed, as reported in **Figure S2a**.[16,17] According to this simulation the formation of an atomic layer of copper oxide around the Cu NCs *ca* 0.2 nm corresponds to a shift in the plasmon resonance peak of around 20 nm. Therefore, an optimized amount of H_2O_2 was introduced in the Cu NCs to target a shift of around 20 nm, shifting from an initial LSPR peak at 556 nm to 574 nm (**Figure S2b**). These modified NCs will be referred as to Cu(H_2O_2) NCs. The UV-Vis spectrum of Cu(H_2O_2) NCs 24 hours after the treatment (red stars) shows no differences with the initial spectra of Cu(H_2O_2) NCs, thus indicating that Cu(H_2O_2) NCs remain stable after the oxidative treatment, which is consistent with the self-limiting nature of the copper oxide.[1] The nature of the copper oxide formed after H_2O_2 treatment was investigated using the Cu LMM Auger spectrum from XPS together with FT-IR (**Figure S2c,d).** Curve fitting of the spectrum revealed a composition corresponding to 46% Cu metal, 36% of $Cu(OH)_2$, 17% of Cu_2O and 0% of CuO (**Figure S2c**).[11] Additionally, the FT-IR spectra comparison between Cu NCs (black) and Cu(H_2O_2) NCs (blue) corroborate the presence of $Cu(OH)_2$ with the appearance of an intense gaussian peak at around 3400 cm^{-1} characteristics of the hydroxyl stretching mode (ν(O-H)), highlighted in blue and of an intense sharp peak at 637 cm^{-1} indicative of Cu-O stretching mode (ν(Cu-O)) while the shoulder at 585 cm^{-1} corresponds to Cu-N stretching mode (ν(Cu-N)), highlighted in green (**Figure S2d**). Altogether, these surface analyses confirmed that the treatment with H_2O_2 forms an ultrathin layer containing hydroxyl groups on the Cu NCs surface and that the Cu(H_2O_2) NCs retain their metallic behaviour and does not alter the native ligands (TOA, TDPA). Additionally, TEM images confirm that the Cu(H_2O_2) remain well spherical with identic size as the as-synthesized Cu NCs (**Figure S2e,f**).

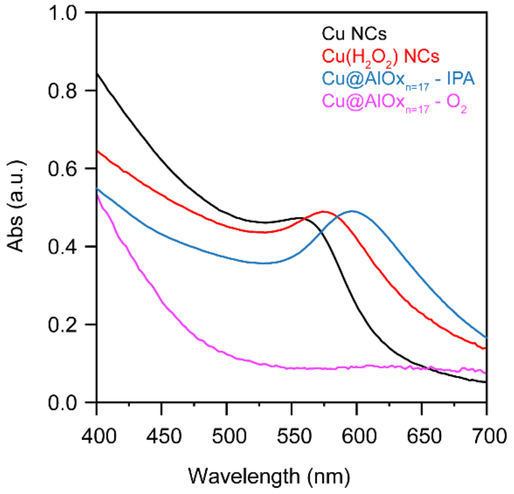

Figure S3. Change in LSPR of c-ALD grown AlOx using O_2 versus IPA as oxygen source on Cu NCs and referred to as Cu@AlOx$_{n=17}$ – O_2 and Cu@AlOx$_{n=17}$ – IPA.

Figure S3 evidences that the use of O_2 gas as oxygen source for the c-ALD, as done in previous publications[2-4], fully oxidize the as-synthesized Cu NCs to Cu_2O NCs, which is indicated by the loss of the plasmonic feature in the UV-VIS. Switching from O_2 to a less oxidizing reagent as the oxygen source, in this case isopropanol, allowed us to preserve the metallic feature of Cu while creating surface hydroxides needed to anchor the alumina shell on the surface.

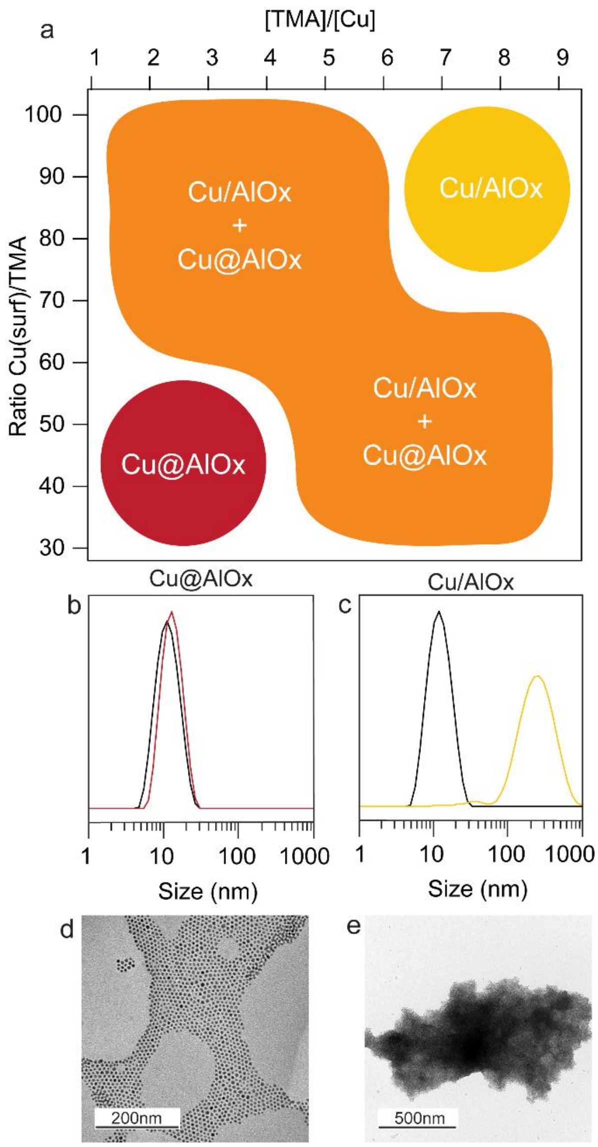

Figure S4. Optimization of the first cycle of c-ALD (i.e. nucleation step) to grow an AlOx shell on Cu NCs. a, Schematic representation of the hybrid configuration obtained as a function of the synthetic parameters used. **b and c**, Representative DLS curves of core @shell Cu@AlOx (**c**, red curve) and of metal/metal-oxide support Cu/AlOx (**d**, yellow curve) compare with as synthesized Cu NCs (black curve). Colors of the DLS curves correspond to representative samples selected from the regions reported in **a**. **d and e**, representative BF-TEM images of Cu@AlOx (**d**) and of Cu/AlOx (**e**).

The outcome of the c-ALD can be tuned between Cu/AlOx metal/support structures or Cu@AlOx core@shell NCs (**Figure S4a**). The synthetic parameters which account for switching from one to the other morphology are the ratio between the concentration of TMA and the concentration of the Cu(H_2O_2) NCs and the number of TMA equivalents injected in the solution (which is referred to in the graph as Cu_{surf}/TMA where Cu_{surf} is the number of Cu

atoms on the NC surface). **Figure S4b-e** evidences that the c-ALD should be operated with both synthetic parameters in their lower range to form a solely core@shell population.

We explain this result as it follows. The binding of TMA molecules on the surface (via protic exchange with surface hydroxyl) competes with the native ligands. A careful balance needs to be established during the nucleation between the anchoring of TMA on the surface of the $Cu(H_2O_2)$ NCs and the stripping of the native ligands, because the latter would cause loss of the colloidal stability and precipitation. The Cu/AlOx metal/support structures are obtained when this balance is not fulfilled and the colloidal stability is lost. Both concentrations of TMA and NCs also affect the conservation of the colloidal stability. A "diluted system" is needed to ensure the homogenous distribution of added TMA around each NC and prevent the encapsulation of more NCs in a single shell (for example).

During the growth, the delicate balance between adding enough TMA to grow the shell while preserving colloidal stability is achieved by adding oxygen-containing ligands such as oleic acid that preserve the colloidal stability while serving as anchoring points for TMA as previously demonstrated by Segura Lecina *et al*.[5]

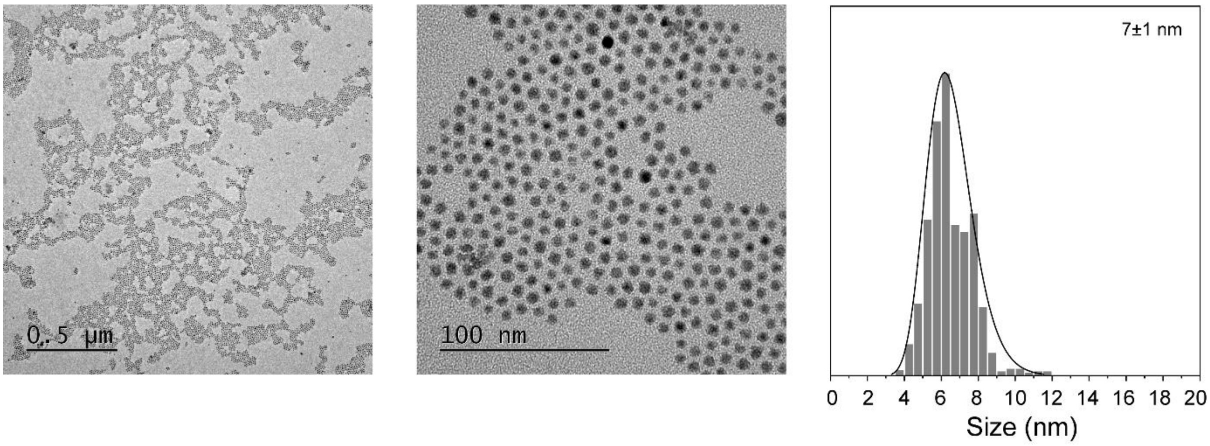

Figure S5. BF-TEM images of Cu@AlOx$_{n=17}$ and corresponding particle size distribution.

The low contrast of the hybrid AlOx shell does not allow to evaluate the shell thickness, thus only the Cu core contributes to the size measurement.

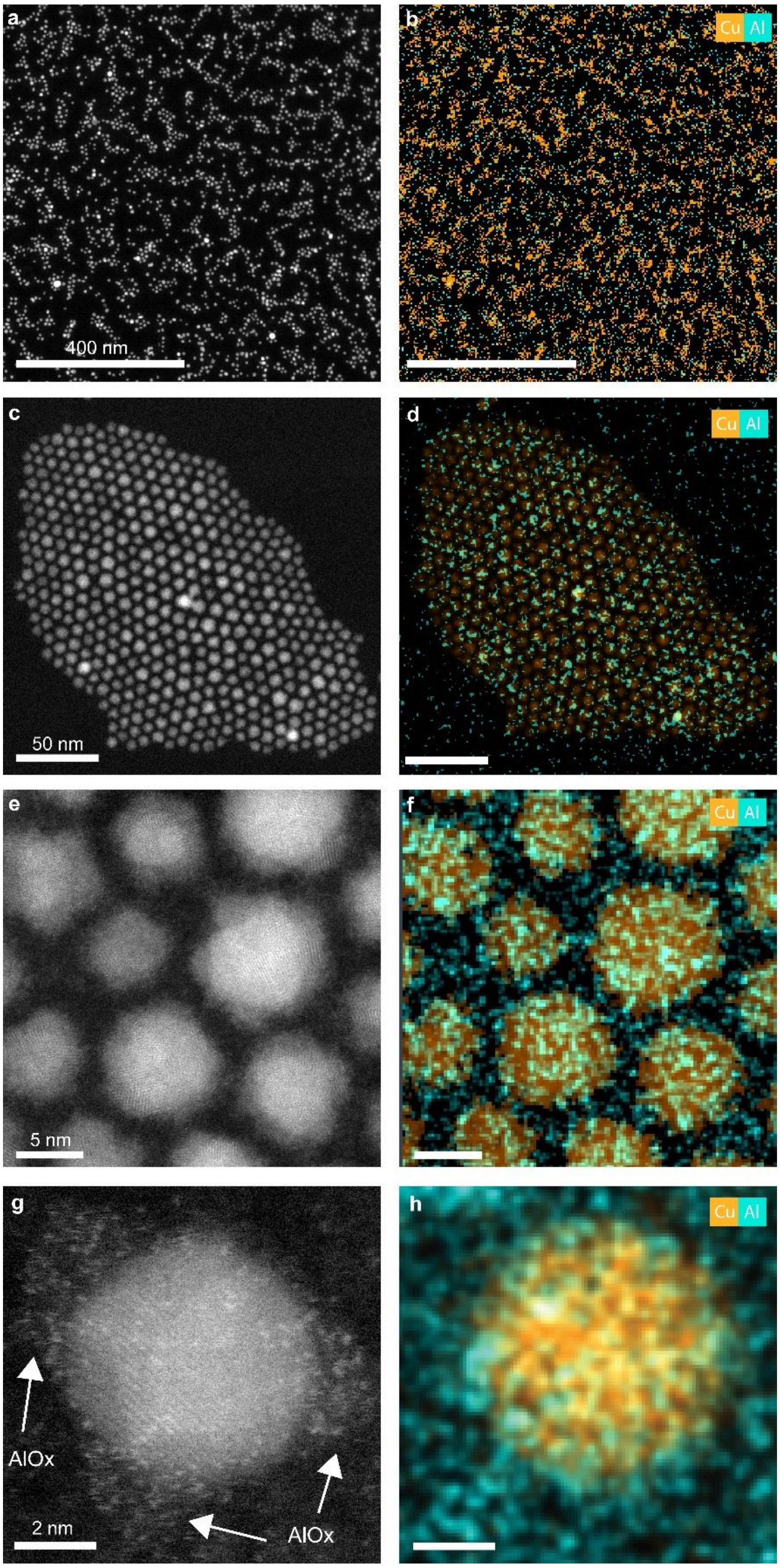

Figure S6. HAADF-STEM images together with EDX elemental map of Cu@$AlOx_{n=17}$ NCs.

The EDX elemental maps show good spatial correlation between the Cu and Al signal at different magnifications. Additionally, the STEM image in Fig S6g evidences the presence of an amorphous alumina shell indicated by white arrows, surrounding the crystalline core of copper. We note that the sample degrades during EDX acquisition, most likely due to the organic ligands embedded in the organic/inorganic shell, thus higher quality analysis is not possible at the moment.

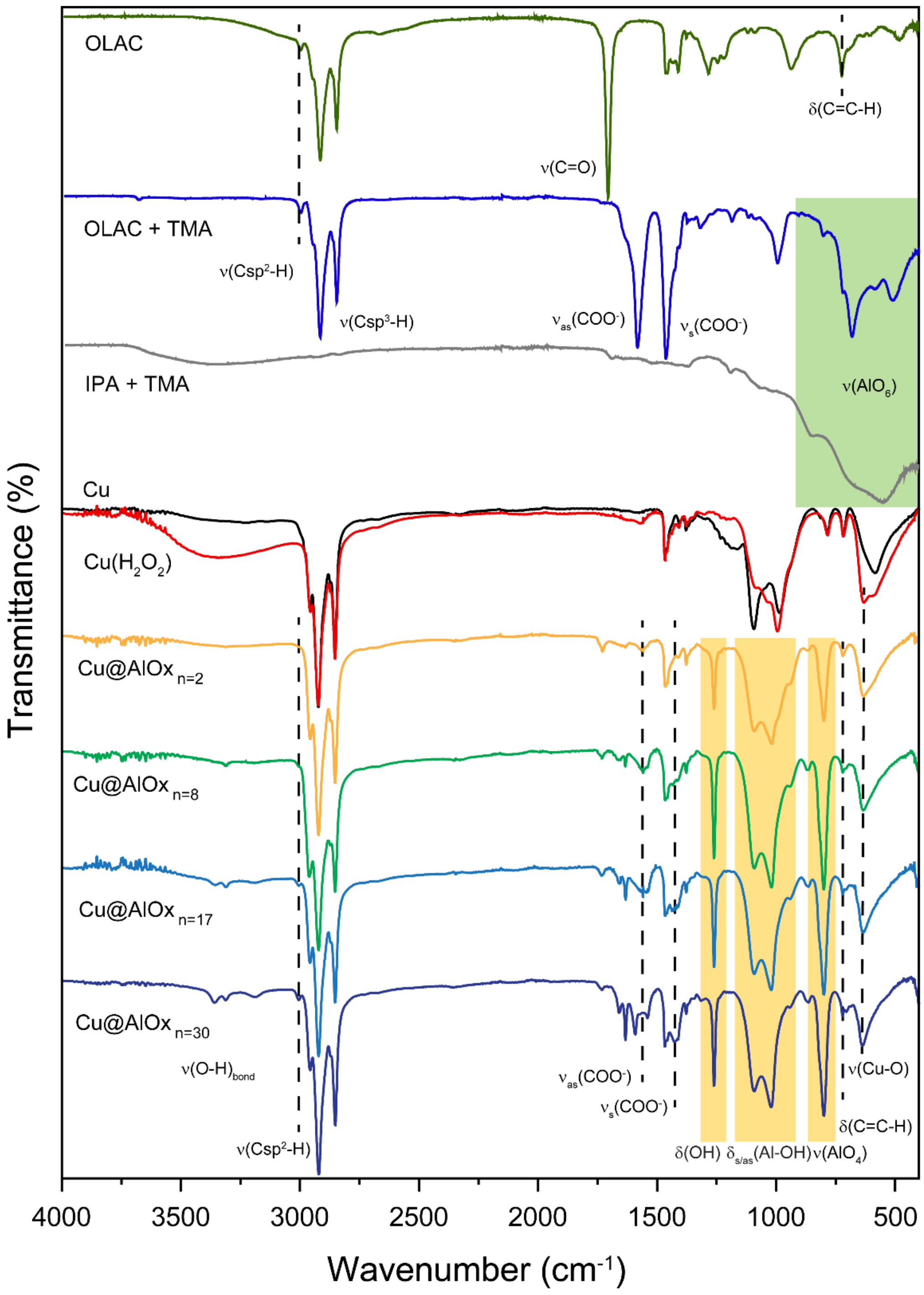

Figure S7. Full-range FT-IR spectra of the as-synthesized Cu NCs (grey), Cu(H_2O_2) NCs (red) and Cu@AlOx$_{n=x}$ with n = 2, 8, 17 and 30 (blue) with AlOx characteristic bands highlighted in yellow, $\nu_{as}(COO^-)$ and $\nu_s(COO^-)$ indicated by dash lines and the stretching ($\nu(Csp^2$-H)) and bending ($\delta(Csp^2$-H, cis) modes of the alkene moieties contain in OLAC, added during c-ALD, highlighted in grey.

The data in Figure S7 show that new bands at 1260, 1095, 1025 and 800 cm^{-1}arise as soon as the c-ALD process starts already in Cu@AlOx$_{n=2}$. These bands are attributed to δ(O-H),

δ_s(Al-OH), δ_{as}(Al-OH) and $\nu(AlO_4)$, respectively. Additionally, three sharp bands at 3360, 3316 and 3192 cm^{-1} emerge in the hydroxyl stretching region and these bands become more and more well defined as the shell grow. The position of these hydroxyls matches well with the boehmite AlOOH structure, as referenced in the main text.[18] Indeed, the other structures of alumina display hydroxyl stretching bands around 3700 cm^{-1}. Interestingly, the boehmite structure is generally defined as edge shared octahedra coordinated Al^{3+} (AlO_6) with a $\nu(AlO_6)$ at 600 cm^{-1}. This feature is observed in the alumina obtained by reacting TMA with IPA or with OLAC as well as in the Cu/AlOx supported structure (Fig. S18). In contrast, the FT-IR of the Cu@AlOx NCs suggest the growth of an amorphous/boehmite-like alumina structure dominated by AlO_4. These structural peculiarities reveal that c-ALD generates a specific and unique structure of alumina around the Cu NCs which deserves future investigation to be fully elucidated.

In addition to the inorganic contribution from the shell, new bands from the OLAC added during the shell growth are observed. The stretching ($\nu(Csp^2$-H)) and bending ($\delta(Csp^2$-H, cis) modes of the alkene moieties emerge along with the two bands at 1566 and 1416 cm^{-1} which correspond to $\nu_{as}(COO^-)$ and $\nu_s(COO^-)$, respectively. The spacing of 150 cm^{-1} between the two bands corresponds to a bridging coordination mode to the alumina, thus confirming that the ligands are entrapped into the alumina matrix conferring the hybrid organic/inorganic nature of the coating.

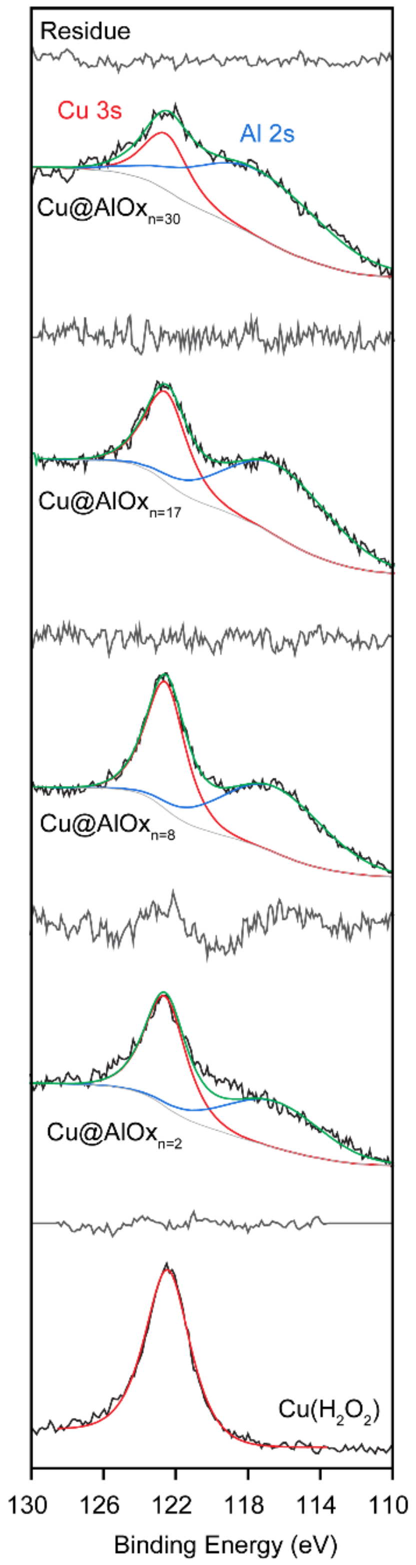

Figure S8. XPS Spectra of Cu 3s/Al 2s region (black) with their respective fits in red and blue and the corresponding envelope (green) and residues (light grey) from the fit, of Cu(H_2O_2) and Cu@AlOx$_{n=x}$ for n=2, 8, 17 and 30. Cu 3s/Al 2s region was selected as region of interest as it is the only region which minimize the overlap between Al and Cu.

Table S1. Detailed XPS peak analysis data from XPS Spectra of Cu 3s/Al 2s region for $Cu(H_2O_2)$ and $Cu@AlOx_{n=x}$ for n=2, 8, 17 and 30.

Sample	Region	Binding Energy (eV)	FWHM (eV)	Area	Area ratio (%)
**$Cu(H_2O_2)$**	Cu 3s	122.44	2.92	6398	100
	Al 2s	\	\	\	\
**$Cu@AlOx_{n=2}$**	Cu 3s	122.56	2.7	2674	52
	Al 2s	116.58	6.25	2427	48
**$Cu@AlOx_{n=8}$**	Cu 3s	122.55	2.57	4244	44
	Al 2s	116.54	6.68	5302	55
**$Cu@AlOx_{n=17}$**	Cu 3s	122.53	2.79	2008	35
	Al 2s	116.27	6.88	3653	65
**$Cu@AlOx_{n=30}$**	Cu 3s	122.46	2.92	2230	30
	Al 2s	117.07	7.00	4989	70

Raw XPS data were fitted using iterated Shirley background subtraction and the position of the C-C bond at core-level C 1s was used to reference the spectra at 284.8 eV. Cu 3s regions were fitted with GL(70) and Al 2s with GL(20) from CasaXPS as metal have an higher Lorentzian contribution.[11,12,19] The Cu 3s region displays the expected values for all samples. Instead, the Al 2s region of all samples shows a particularly large FWHM (>4eV) which indicates the presence of different coordination environments of Al^{3+}, which is in line with the FT-IR data indicating an amorphous/boehmite structure. Indeed, the overlapping of different Al^{3+} signals (Al^{IV}, Al^{V} and Al^{VI} coordination) generate line broadening.[20]

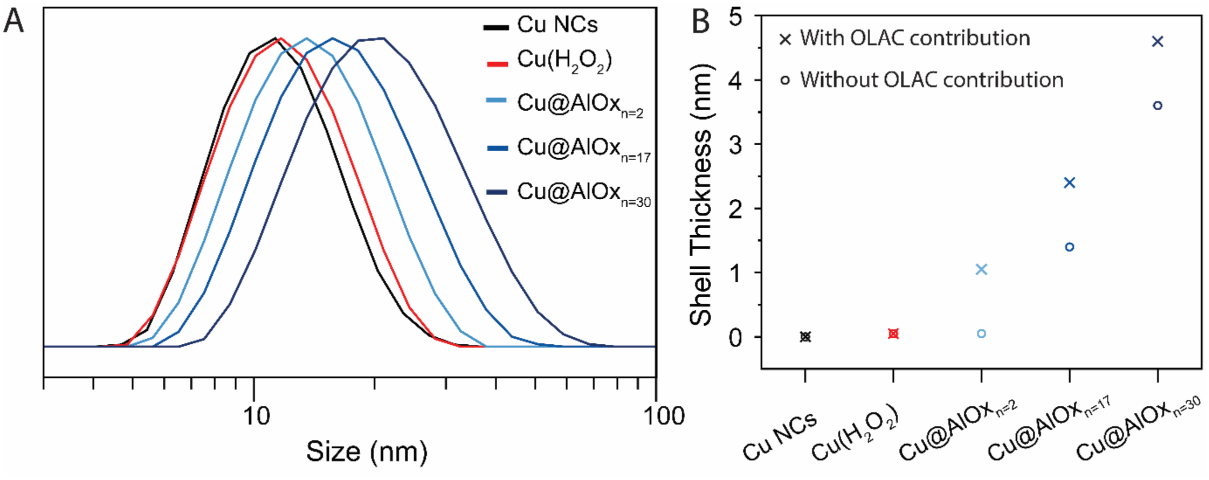

Figure S9. DLS characterization. A. DLS curves in intensity as function as the number of c-ALD cycles. **B.** Shell thickness extracted from DLS curve via a lognormal fitted model with and without OLAC contribution

Figure S9 displays the DLS curves obtained for Cu@AlOx at different cycles (A) and the shell thickness extracted as function of the number of c-ALD cycles (B). We provide two different values for the shell thickness, one which includes the contribution of OLAC added during the c-ALD and one where around 1 nm was subtracted to account for the presence of the OLAC molecule and to get a more representative thickness of the alumina coating itself.

Supplementary Note 1:

Considering spherical Cu NCs, the volume (V_{sphere}) and surface (A_{sphere}) of a single Cu sphere are:

$$V_{sphere} = \frac{4}{3} \times \pi \times \left(\frac{d}{2}\right)^3 \tag{1}$$

$$S_{sphere} = 4 \times \pi \times \left(\frac{d}{2}\right)^2 \tag{2}$$

With d being the average diameter of the spherical Cu NCs, obtained by TEM. The substitution of d= 7 nm gives:

$$\begin{aligned} V_{sphere} &= \frac{4}{3} \times \pi \times \left(\frac{7}{2}\right)^3 \\ &\approx 180\ nm^3 \end{aligned} \tag{3}$$

$$\begin{aligned} A_{sphere} &= 4 \times \pi \times \left(\frac{7}{2}\right)^2 \\ &\approx 155\ nm^2 \end{aligned} \tag{4}$$

Cu possess a FCC structure with a lattice constant (a) of 3.62Å and 2 atoms for each surface of the unit cell[21], it gives a surface density of atoms (δ_{Cu}) of[21]:

$$\begin{aligned} \delta_{Cu} &= \frac{2}{a^2} \\ &\approx 15\ atoms.nm^{-2} \end{aligned} \tag{5}$$

Consequently, the number of Cu atoms on the surface of the Cu NCs ($N_{S,Cu}$) can be approximated to:

$$\begin{aligned} N_{S,Cu} &= A_{sphere} \times \delta_{Cu} \\ &= 155 \times 15 \\ &\approx 2500\ atoms\ of\ Cu\ on\ the\ surface \end{aligned} \tag{6}$$

Similarly, the number of Cu atoms in each Cu NCs ($N_{V,Cu}$), considering a total number of atoms of 4 per unit cell, can be approximated to:

$$N_{V,Cu} = \frac{4 \times V_{sphere}}{a^3} \tag{7}$$

$$= \frac{4 \times 180}{(0.362)^3} \approx 13500 \; atoms\ of\ Cu\ in\ each\ NCs$$

Consequently, the atomic ratio surface to volume $x_{S,V}$

$$x_{S,V} = \frac{N_{S,Cu}}{N_{V,Cu}} \tag{8}$$

$$= \frac{2500}{13500}$$

$$\approx 20\%$$

The same surface-to-volume atomic ratio is obtained if the calculations are performed considering an average Van Der Walls radius of Cu atoms of 140 pm instead of the atomic density from the FCC structure.

It is noted that this atomic ratio of 20% should be interpreted just as a rough estimate. Indeed, Cu NCs are not perfect spheres but rather faceted NCs with surface defect, thus a higher atomic ratio can be expected.

Considering 2500 atoms of Cu on the surface of each NC, the mass ratio (r) of Cu:Al for a monolayer coverage can be estimated as follow:

$$r = \frac{m_{Al}}{m_{Cu}} \tag{9}$$

Assuming a monolayer of Al_2O_3, the Al atoms represent 2/5 of the atoms constituting the monolayer that is, 2/5 of 2500 atoms, so:

$$m_{Al} = \frac{2}{5} \times 2500 \times \frac{M_{Al}}{N_A} \tag{10}$$

with m_{Al} the mass of aluminum, M_{Al} the molar mass of aluminum (27 $g.mol^{-1}$) and N_A the Avogadro constant ($6{,}02.10^{23}$ mol^{-1}).

and:

$$m_{Cu} = V_{sphere} \times d_{Cu} \tag{11}$$

with m_{Cu} the mass of one sphere of Cu and d_{Cu} the density of copper (8.96 $g.cm^{-3}$).

Therefore:

$$r = \frac{2 \times 2500 \times M_{Al}}{5 \times V_{sphere} \times d_{Cu} \times N_A} \tag{12}$$

$$= \frac{2 \times 2500 \times 27}{5 \times 180 \times 8.96 \times 6.02 \times 10^2}$$

$$\approx 3\%$$

A Cu:Al mass ratio of 3% is exactly that added in $Cu@AlOx_{n=17}$, so the calculation above confirms that full encapsulation of the Cu NCs can indeed be expected for this number of cycles.

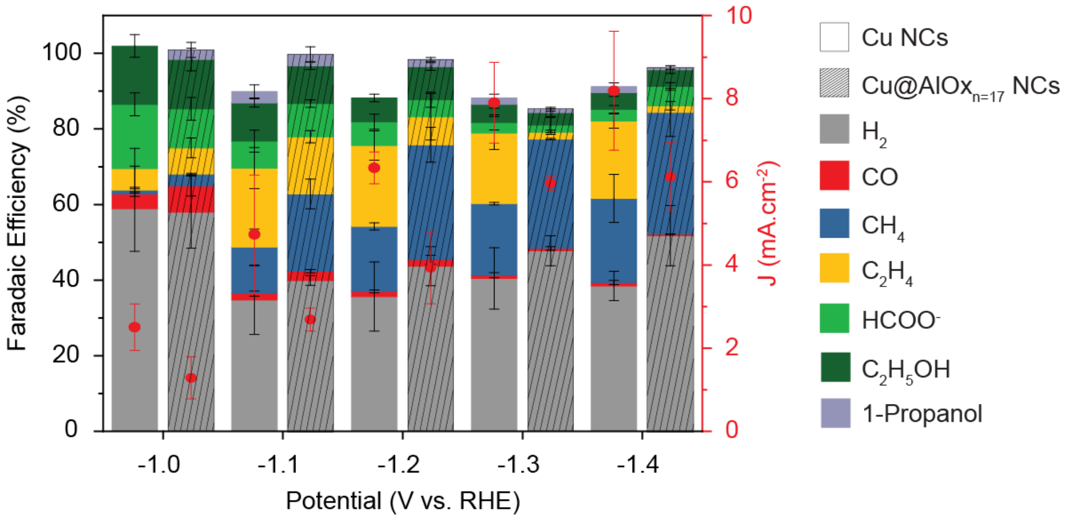

Figure S10. Comparison of the CO_2RR performance of Cu NCs and of Cu@AlOx$_{n=17}$ NCs. Total FEs for all gaseous products (i.e., H_2, CO, CH_4, C_2H_4) and the main liquid products (i.e., $HCOO^-$, C_2H_5OH, 1-propanol) together with the geometric current density (red, right axis) as a function of applied potential. H_2 FE remain at similar level in both cases and only some reduction of the current is observed. The data are the average of three independent experiments and the error bars are the calculated standard deviation.

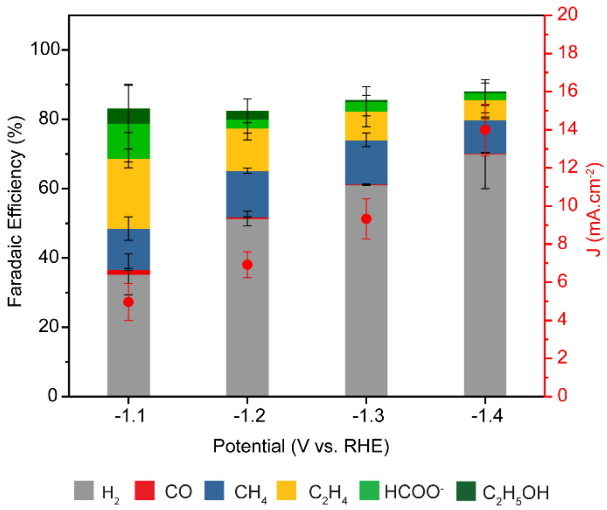

Figure S11. Overall CO_2RR performance of Cu(H_2O_2) NCs. a, Total FEs for all gaseous products (i.e., H_2, CO, CH_4, C_2H_4) and the main liquid products (i.e., $HCOO^-$, C_2H_5OH) together with the geometric current density (red, right axis) as a function of applied potential. The data are the average of three independent experiments and the error bars are the calculated standard deviation.

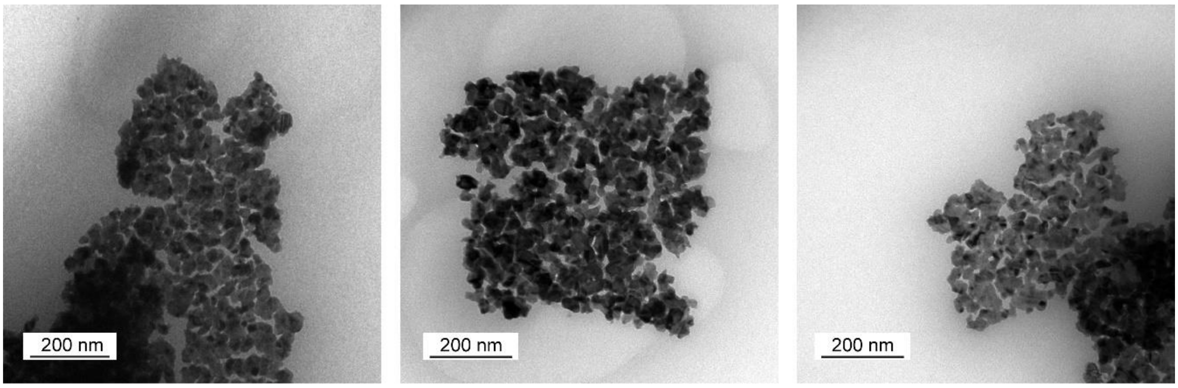

Figure S12. Representative BF-TEM images of Cu(H_2O_2) NCs after 1 hour of CO_2RR at -1.1 V vs RHE.

The Cu(H_2O_2) NCs exhibit the same current density and product distribution of the as-synthesized Cu NCs at -1.1V vs RHE (Figure S11 and Table S2). However, the current density and hydrogen production increase compared to as-synthesized Cu NCs at more negative potentials. We note that the Cu(H_2O_2) NCs reconstructs, similarly to the as-synthesized Cu NCs. However, a qualitative interpretation of the TEM images after CO_2RR indicates more extended copper domains compared to those observed in the as-synthesized Cu NCs. The presence of bigger Cu grains can explain the higher current density and hydrogen production measured at more negative potentials (Figure S11 and Table S2).

Table S2. FEs of as-synthesized Cu NCs, Cu(H_2O_2) and Cu@AlOx$_{n=17}$ NCs for the main gaseous and liquid products averaged over one hour of CO_2RR.

As-synthesized Cu NCs

V vs RHE	H_2	CO	CH_4	C_2H_4	$HCOO^-$	C_2H_5OH	C_3H_7OH	Current ($mA.cm^{-2}$)
-1.1	34.7±9.1	1.7±0.7	12.3±4.8	20.8±5.4	7.2±2.9	10.1±1.1	3.1±1.7	4.7±1.4
-1.2	35.7±9.1	1.4±0.4	17.2±0.9	21.4±3.9	6.3±2.0	6.3±1.0	0.0	6.3±0.4
-1.3	40.4±8.1	0.9±0.7	19.0±0.3	18.7±4.4	2.8±2.0	4.8±1.0	1.7±1.0	7.9±1.0
-1.4	38.5±3.9	0.9±0.6	22.3±6.3	20.5±1.9	3.1±1.0	4.4±1.0	1.6±1.0	8.2±1.4

Cu(H_2O_2) NCs

V vs RHE	H_2	CO	CH_4	C_2H_4	$HCOO^-$	C_2H_5OH	C_3H_7OH	Current ($mA.cm^{-2}$)
-1.1	35.3±5.9	1.4±0.3	11.8±3.4	20.2±2.7	10.2±1.3	11.2±4.1	6.8±1.1	4.9±1.0
-1.2	51.4±2.1	0.4±0.1	13.4±0.7	12.3±1.5	2.5±1.8	5.9±2.3	0	6.9±0.7
-1.3	61.1±11.3	0.2±0.1	12.8±2.0	8.3±4.5	2.8±1.6	4.2±0.3	0	9.3±1.1
-1.4	70.7±10.1	0.2±0.1	9.6±2.0	5.7±4.9	2.1±0.8	3.7±0.2	0	13.9±1.4

Cu@AlOx$_{n=17}$ NCs

V vs RHE	H_2	CO	CH_4	C_2H_4	$HCOO^-$	C_2H_5OH	C_3H_7OH	Current ($mA.cm^{-2}$)
-1.1	39.9±1.3	2.4±0.4	20.5±3.9	15.1±1.6	8.8±1.0	10.0±1.0	3.1±2.1	2.7±0.3
-1.2	43.6±5.2	1.7±1.2	30.4±4.6	7.4±6.2	4.5±2.0	8.8±1.0	0.0	3.9±0.9
-1.3	47.7±4.0	0.6±0.5	29.0±0.2	0.6±1.9	1.9±2.0	3.3±2.0	1.7±0.5	5.9±0.2
-1.4	51.7±8.0	0.4±0.1	32.3±6.4	1.1±5.1	5.1±1.0	4.4±1.5	1.6±0.5	6.1±0.8

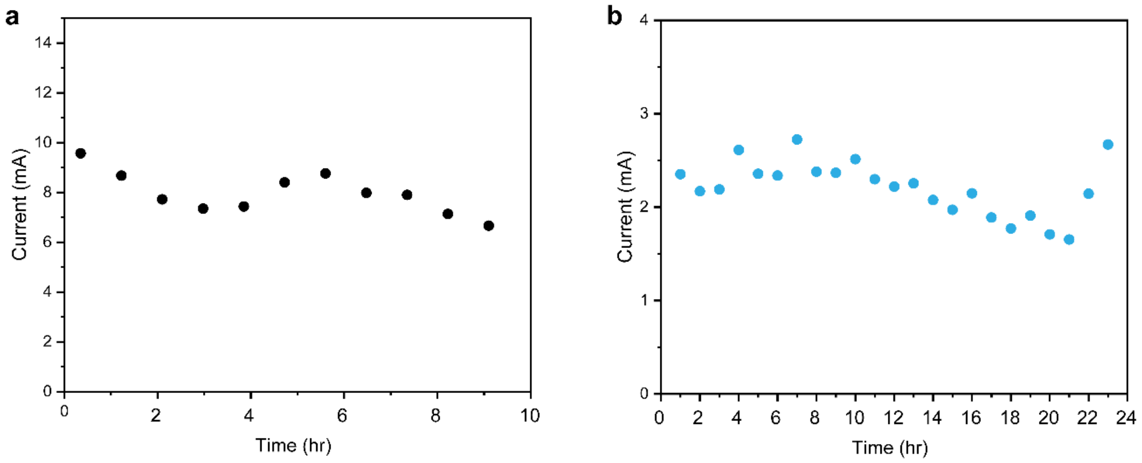

Figure S13. Long chronoamperometry test. a,b. Total current density, including HER and CO2RR, for Cu NCs (**a**) and Cu@AlOx$_{n=17}$ NCs (**b**) at -1.1 V vs RHE in 0.1M $KHCO_3$ in a H-cell. Methane selectivity was chosen as metric for reactive stability in Fig. 2 because of its correlation to the reconstruction of the Cu NCs.

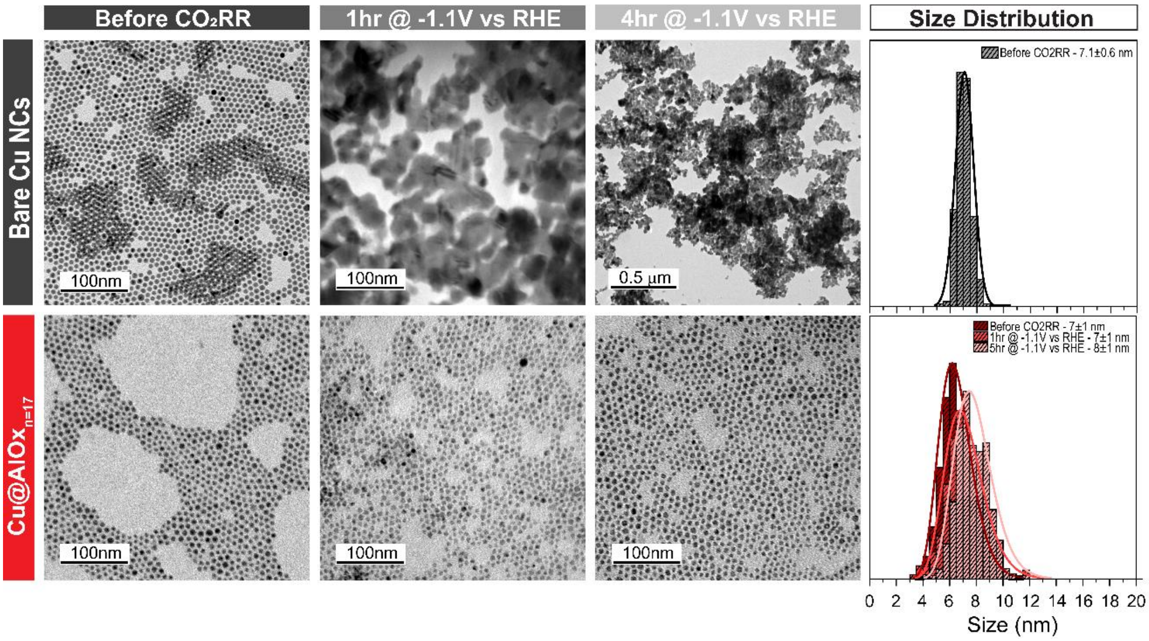

Figure S14. Top row: Representative BF-TEM images of Cu NCs before and after 1 hour and 4 hours of CO_2RR at -1.1 V vs RHE together with the size distribution of the as-synthesized NCs. Bottom row: BF-TEM images of Cu@AlOx$_{n=17}$ before and after 1 hour and 4 hours of CO_2RR at -1.1 V vs RHE together their corresponding NC size distribution showing no change within the standard deviation of the measurement.

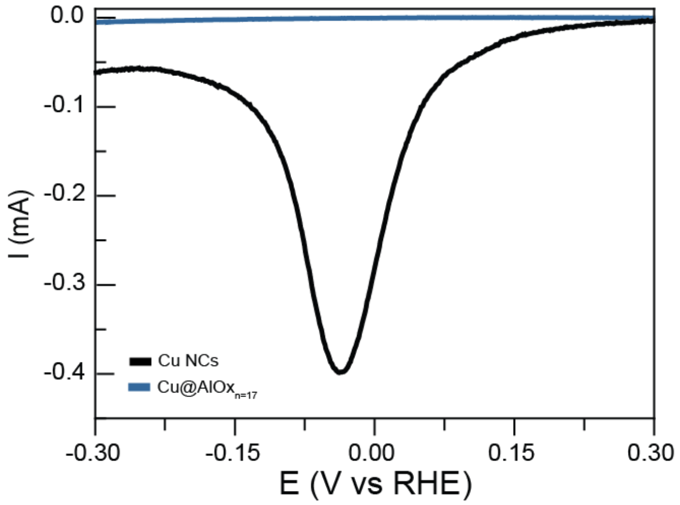

Figure S15. Zoom-in of the linear sweep voltammogram from OCP to -1.1 vs RHE measured for the as-synthesized Cu NCs and the Cu@AlOx$_{n=17}$ NCs.

Complementary insight into the morphological stability of the shell were retrieved from pre-CO_2RR linear sweep voltammetry (LSV). The potential window displayed in the figure focuses on the electro-stripping region of the native ligand, which is around 0 V vs RHE based on previous studies[2]. As expected, the ligands are completely electro-stripped from the surface of the as-synthesized Cu NCs as a clear peak appears. No electro-desorption is observed for the Cu@AlOx sample. This result provides additional support to the fact that the ligands and the alumina shell remain bound to the surface of the NCs under cathodic potential and during CO_2RR, which is in line with the ligand locking induced by the c-ALD grown oxide shells previously reported.[4]

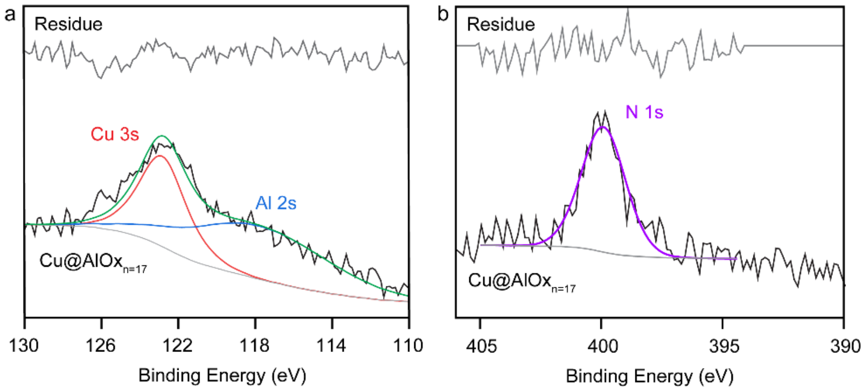

Figure S16. Post 4 hours CO_2RR XPS spectra. a, Cu 3s/Al 2s region (black) with their respective fits in red and blue and the corresponding envelope (green) and residues (light grey). **b,** N 1s region with fit (violet) and residue (light grey). The contribution of alumina and nitrogen containing ligands (TOA) is cleary detected, thus reinforcing the conclusion that the alumina shell with its embedded ligands is stable during CO_2RR.

Table S3. Detailed XPS comparison of Cu 3s/Al 2s region for Cu@AlOx$_{n=17}$ before and after 4 hours of CO_2RR at -1.1 V vs RHE.

Sample	Region	Binding Energy (eV)	FWHM (eV)	Area	Area ratio (%)
**Cu@AlOx$_{n=17}$** *Before CO_2RR*	Cu 3s	122.55	2.57	4244	44
	Al 2s	116.54	6.68	5302	56
	N 1s	399.7	1.5	895	/
**Cu@AlOx$_{n=17}$** *After 4 hours CO_2RR*	Cu 3s	122.78	2.9	1576	46
	Al 2s	117.39	7	1865	54
	N 1s	399.9	1.5	1227	/

Raw XPS data were fitted using iterated Shirley background subtraction and the position of the C-C bond at core-level C 1s was used to reference the spectra at 284.8 eV. Cu 3s regions were fitted with GL(70) and Al 2s with GL(20) from CasaXPS as metal have an higher Lorentzian contribution.[11,12,19]

The Al peak shifts toward higher binding energy (BE) after CO_2RR which indicates more oxidized/electron depleted Al cations. We hypothesize that this change is correlated to the changes in overall copper oxidation state during CO2RR, which are commented on later in the manuscript and herein.

Before CO_2RR, Cu^{+2} is the dominant speciation of copper on the surface. CuO can be regarded to as a Lewis basic oxide, thus its presence results into more electron rich Al cations. Indeed, the Al^{3+} signal is located at lower BE compared to our previous work (116.5 vs 119.2 eV).[3-5]

During CO2RR, Cu^0 becomes the dominant surface speciation with fewer remaining Cu^{2+} (only those bound to the alumina network). As the Cu^{+2} contribution decreases, the Al cations become less electron rich, which results in a shift toward higher binding energy in the XPS.

The fact that the FT-IR does not exhibit change before/after CO_2RR (Fig. 2f) and that no change in the FWHM in the Al 2s region is observed are consistent with no structural or chemical change of the AlOx occurring upon CO_2RR. As commented later, the CVs do not indicate any major change in the Cu@AlOx either (Fig.S29).

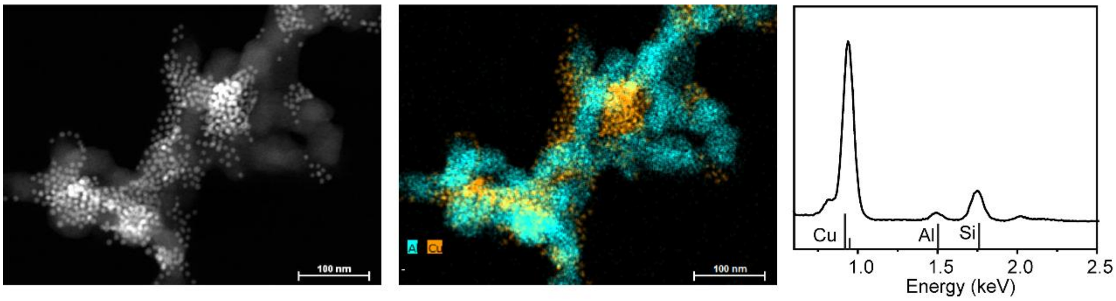

Figure S17. A, B Representative HAADF-STEM image of Cu/AlOx and corresponding EDX elemental map. **C.** Corresponding EDX Spectrum.

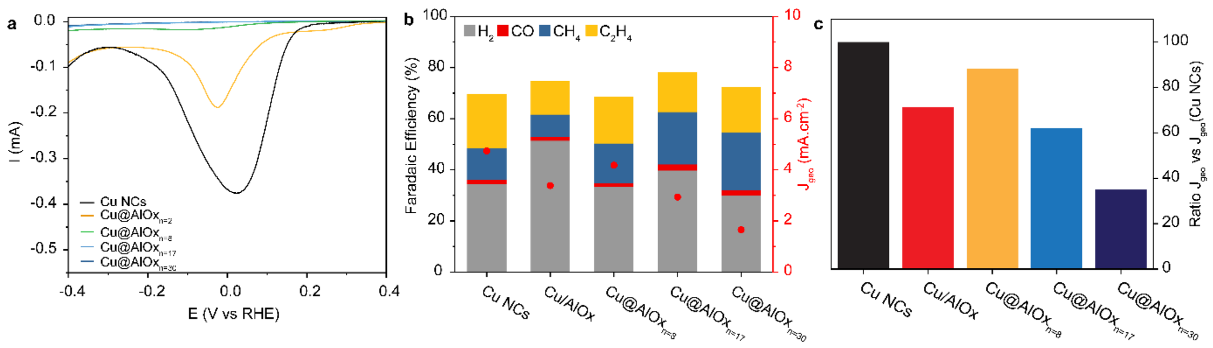

Figure S18. Evaluation of the CO2RR performance for Cu@AlOx NCs at varied c-ALD cycles (n) and for metal/oxide support Cu/AlOx NCs. **a,** LSV for Cu@AlOx$_{n=x}$ with n=2, 8, 17 and 30. **b,** FEs for all gaseous products (i.e., H_2, CO, CH_4, C_2H_4) for as-synthesized Cu NCs, Cu/AlOx and Cu@AlOx$_{n=x}$ with n= 8, 17 and 30. **c,** Ratio of geometric current density of all samples compared to the geometric current density of Cu NCs

The LSV data (Fig S18a) show that ligands detachment occurs for the as-synthesized Cu NCs and for Cu@AlOx samples with n<17 when the ligands detachment peak does not appear. This result implies that the hybrid alumina shell is not stable for n<17. Concomitantly to the shell stability, methane becomes the dominant product for Cu@AlOx$_{n=x}$ with n= 17 and 30 (Fig S18b). Overall, the geometric current density decreases as n increases (Figure S18d).

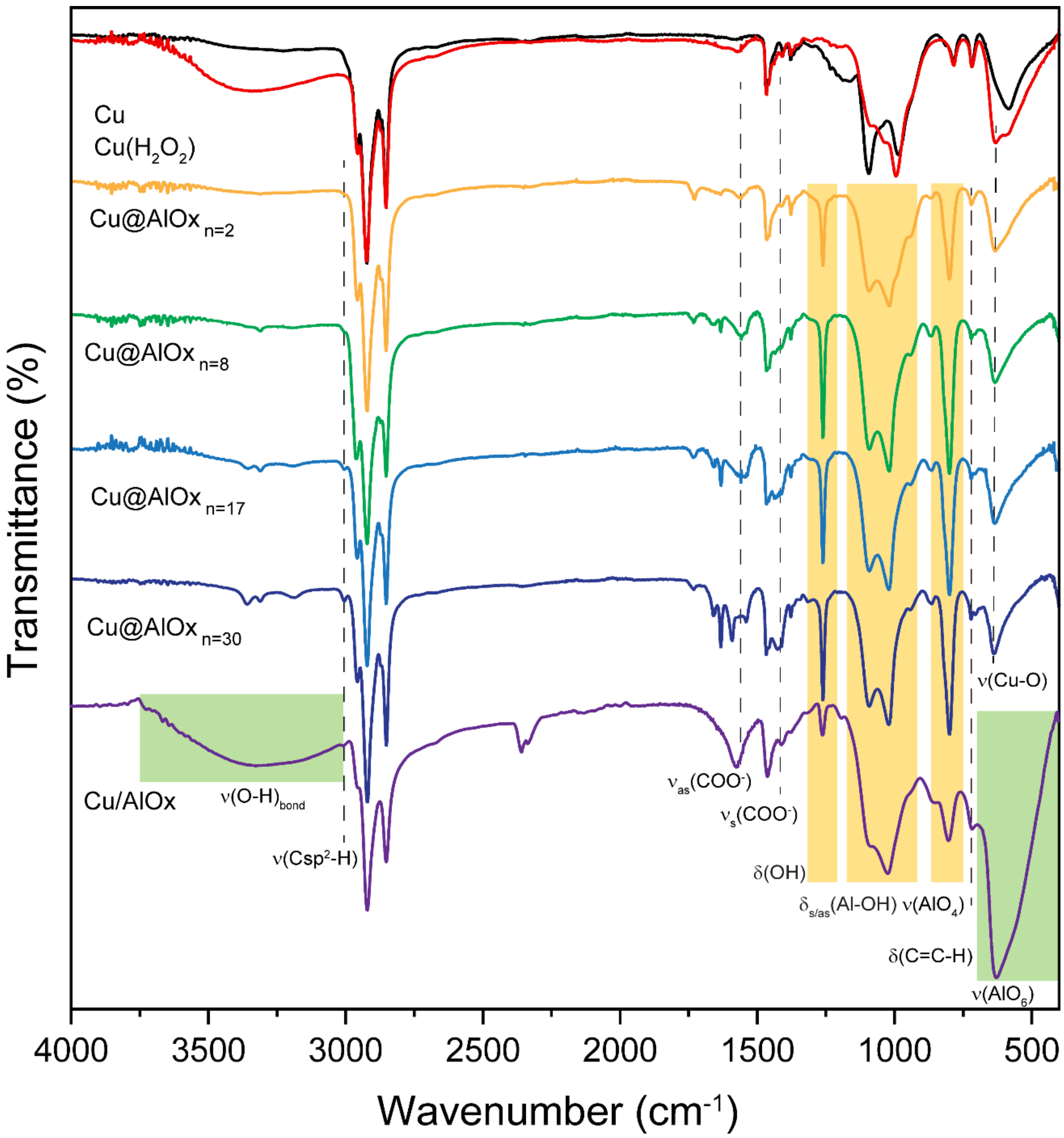

Figure S19. FT-IR spectra of the Cu/AlOx compared with Cu@AlOx showing the predominance feature of AlO_6 and bonded OH characteristic of Brønsted acid oxide.

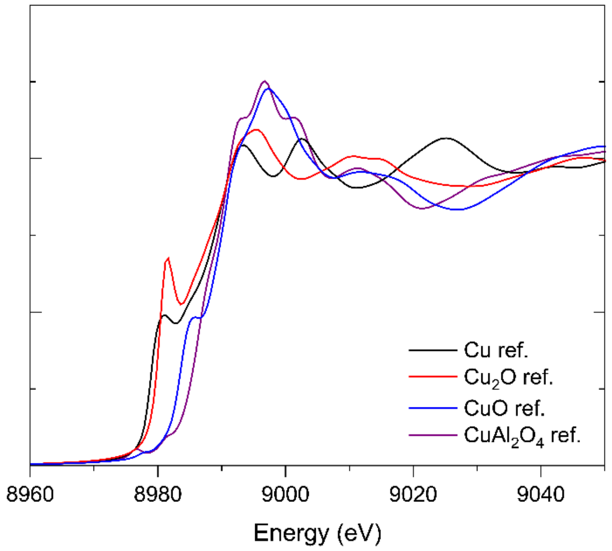

Figure S20. Cu K-edge XANES spectra of Cu, Cu_2O, CuO and $CuAl_2O_4$ commercial references.

Commercial Cu foil, Cu_2O, CuO, and $CuAl_2O_4$ were used as standards to build the LCA (Linear Combination Analysis) models $CuAl_2O_4$ was chosen as an additional reference for Cu^{2+} to consider the eventual formation of binary oxide between the copper core and the alumina shell, which was not observed.

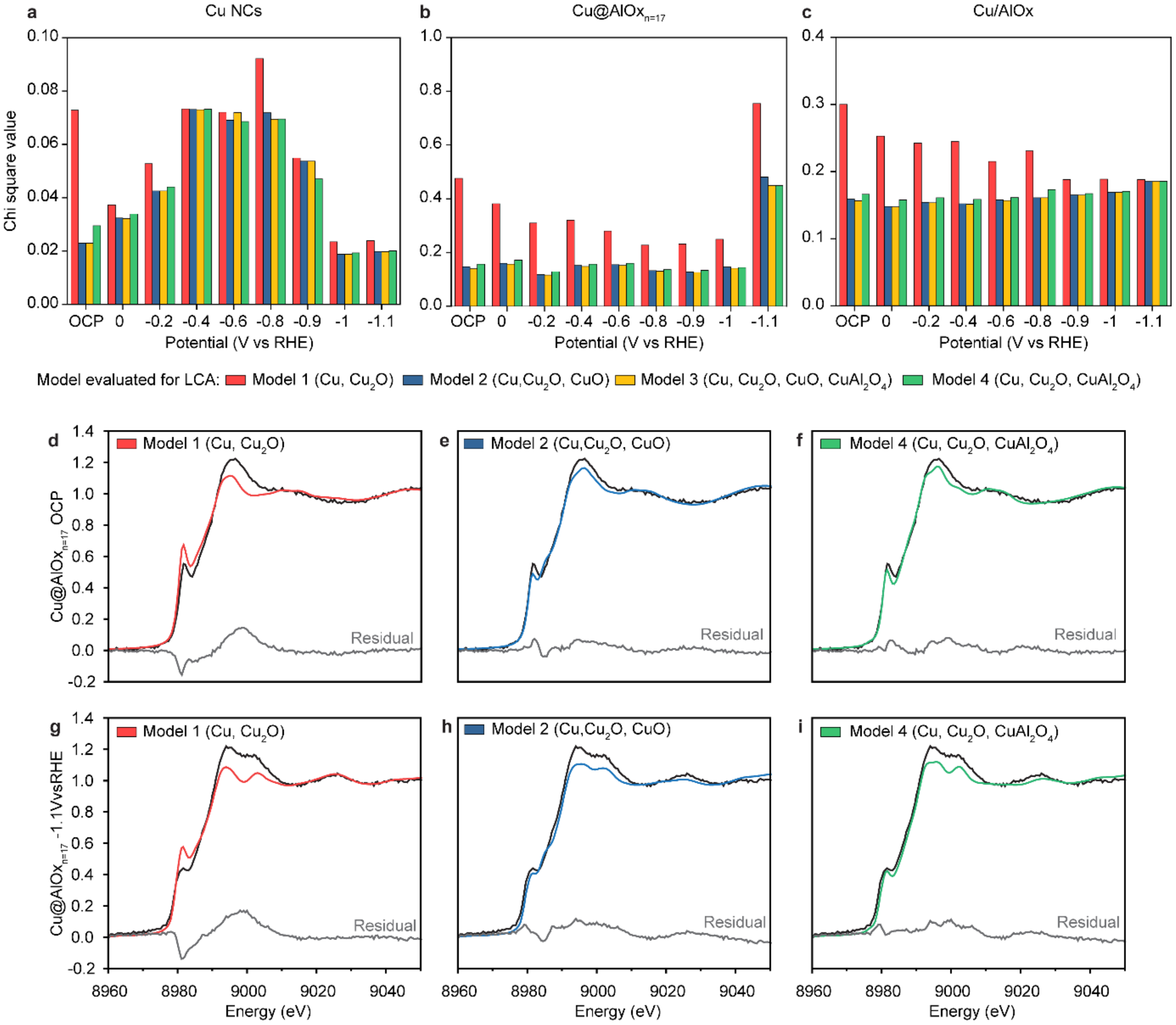

Figure S21. LCA analysis on the XANES spectra. a-c Evolution of Chi square as a function of the applied potential for the three different systems and the four LCA models evaluated based on the bulk standards reported in **Figure S20**. **d-i** As-acquired XANES spectra together with the fitted model and the residual for Cu@AlOx$_{n=17}$ at OCP (**d-f**) and at -1.1V vs RHE (**g-i**).

These four LCA models were built by considering different components of copper oxides, as indicated in the legend of the figure.

At OCP, the three systems are best modeled by assuming a mixed of Cu^0, Cu^+ and Cu^{2+}, as a significant decrease in chi square is observed from model 1 to model 2, 3 or 4 (**Fig S21a-c**). **Fig S21d-g** further evidences that only the models considering a Cu^{2+} component more accurately fit the pre-edge and white line feature of the XANES spectra at OCP and at applied cathodic voltage in the case of Cu@AlOx$_{n=17}$, which was selected as a representative case.

Interestingly, the Chi square for the Cu^{2+} fraction is similar when considering either CuO (model 2), or $CuAl_2O_4$ (model 4) or indeed a mixture of the two (model 3). However, additional characterization (XPS, FT-IR, SWV, LSV) exclude Cu-Al alloys or binary oxides, at least for OCP.

This observation indicates that the Cu@AlOx interface, which we indicate as CuO(Al), generated in our catalyst is a rather unique and cannot be adequately captured by the bulk references. This structural uniqueness is later confirmed by EXAFS where one of the two Cu-O scattering peaks could not be accurately fitted nor by CuO or by $CuAl_2O_4$.

As the applied potential becomes more cathodic, the difference between model 1 and the other models becomes statistically insignificant for Cu NCs and Cu/AlOx which indicates that the oxide fractions tend to be fully reduced. However, Cu@AlOx$_{n=17}$ stands as unique as across the entire range of potentials because a mixture of Cu^0, Cu^+ and Cu^{2+} always models the experimental data to a statistically superior degree in these cases (Note: an increase in Chi square occurs for Cu@AlOx$_{n=17}$ when reaching the -1.1 V vs RHE; this increase is due to some catalyst detachment during the experiments but does not alter the integrity of the data). Again, the modeling of the exact nature of Cu^{2+} remain elusive from the bulk standards. The fact that the models 3 and 4 which include $CuAl_2O_4$ system never result in any statistically significant improvement to the fits compared to the model 2 suggests that the formation of this mixed oxide is highly unlikely. Moreover, if Cu-Al alloying or the formation of some Cu-Al binary oxide were taking place, those effects should have been even more pronounced in Cu/AlOx due to the excess of alumina surrounding the Cu NCs, which is not the case. All the above provides us confidence to fairly exclude the formation of $CuAl_2O_4$ and to pick CuO as the most representative model of the Cu^{2+} fraction in the LCA analyses reported in the manuscript.

We note that $CuAl_2O_4$ has been reported in the literature as CO_2RR catalyst generating C_{2+} products.[22–24] Interestingly, the copper speciation during CO_2RR changes from Cu^{2+} to Cu^+ or even Cu^0, which implies a complete chemical and structural change of the material. While the exact reason behind the instability of the Cu^{+2}-O-Al bond is not elucidated, we speculate that similar to our Cu/AlOx, the bond might be more prone to dealumination due to a more Bronsted acid character of the synthesized $CuAl_2O_4$

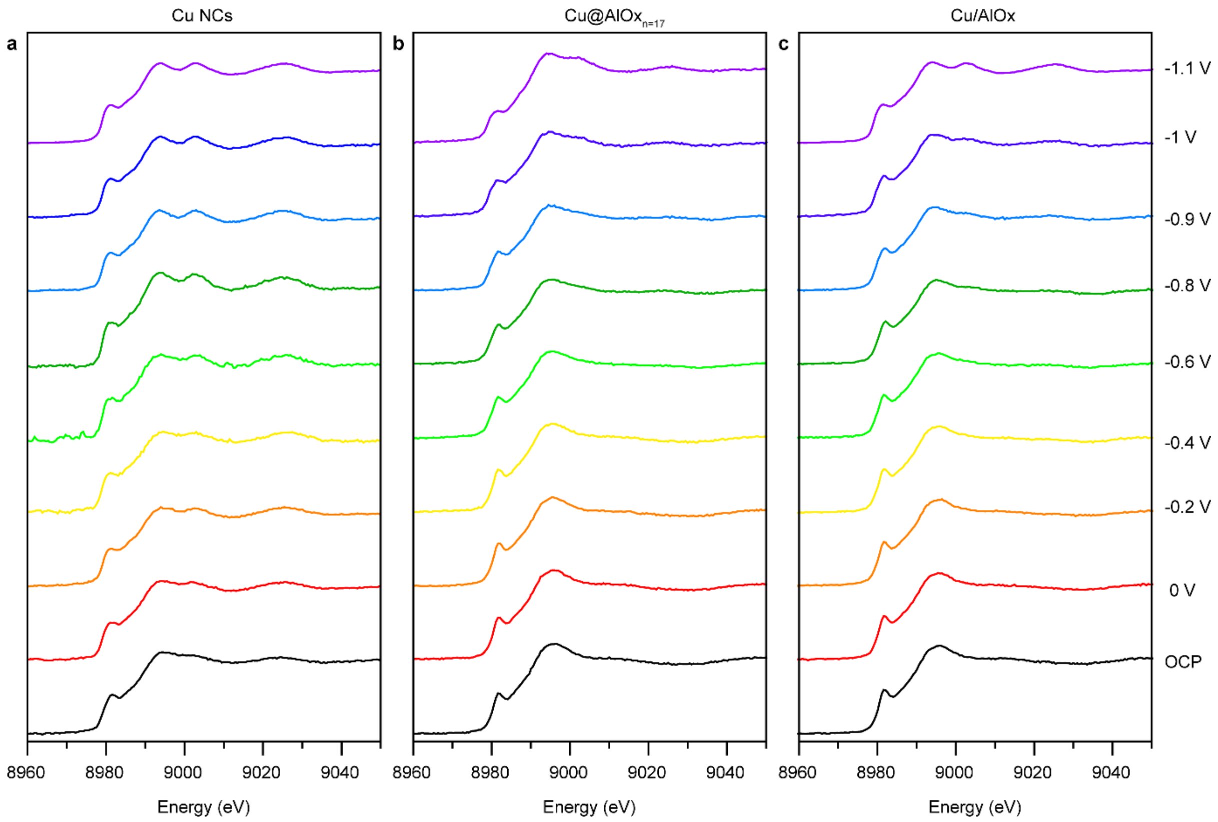

Figure S22. Evolution of operando XANES spectra as function of the applied potentials (vs RHE) for **a,** Cu NCs **b,** Cu@AlOx$_{n=17}$ and **c,** Cu/AlOx.

The evolution of the copper species as a function of the applied potential extracted from LCA for the three systems is summarized in **Figure 4d-f**. **Figure S22** reports the original data. Below we write more detailed comments on the data.

The as-synthesized Cu NCs display $Cu^0/Cu^+/Cu^{2+}$ fractions of around 65/25/10 % at OCP, where the contribution of Cu^+ and Cu^{2+} is attributed to oxidation of the sample upon contact with the electrolyte. Indeed, these Cu spheres were shown to self-passivate with an amorphous layer of CuO surface oxide and Cu_2O core.[25] With a more cathodic potential being applied, the Cu NCs completely reduce to Cu^0 with a contribution of above 90% to the signal and residual Cu^+ below 10% while no Cu^{2+} is found at -1.1V vs RHE. These data are consistent with previous results.[26,27]

At OCP, Cu@AlOx$_{n=17}$ and Cu/AlOx display similar oxide mixture with less than 10% of Cu^0, around 55% Cu^{+1} and around 30% of Cu^{+2}. Cu^{+2} originates from the oxidative pretreatment with H_2O_2 as 30% matches very well with the 36% evaluated from the Cu LMM Auger in Figure S2, which correspond roughly to a monolayer of Cu^{+2} targeted as sufficient

for the alumina nucleation during c-ALD. Instead, the Cu^{+1} is associated with oxidation of the sample upon exposure to the electrolyte similarly to the as-synthesized Cu NCs.[27]

As the applied potential becomes more cathodic, the two systems exhibit a different behavior. $Cu@AlOx_{n=17}$ retains a significant fraction of Cu^{+2} (ca 20%) at -1.1V vs RHE. We propose that the drop from the initial 35 % is a consequence of not all the Cu surface atoms binding to AlOx and would correlate with a porous shell. Cu/AlOx whilst losing completely the Cu^{2+} component, does, however, retains around 35% of Cu^{+1} fraction when reaching -1.1 V vs RHE.

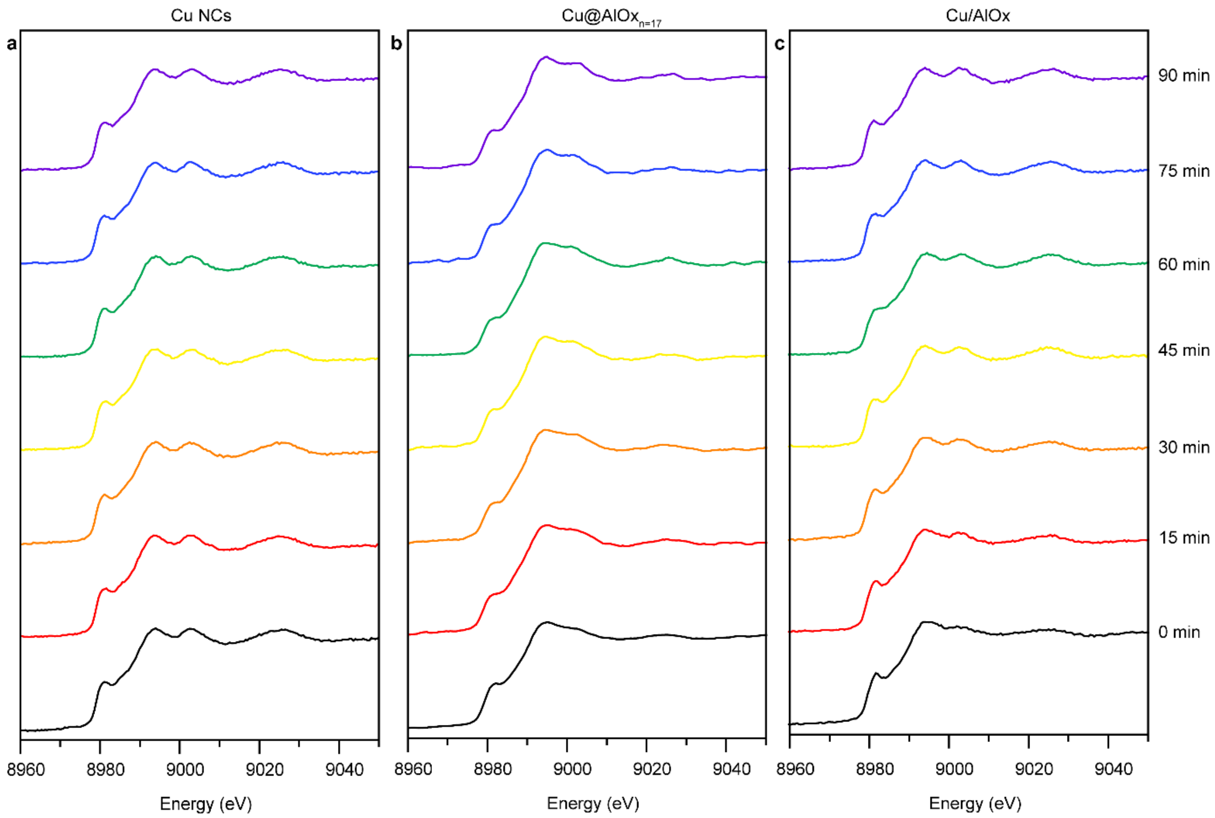

Figure S23. Evolution of operando XANES spectra as function of time at -1.1 V vs RHE applied potential for **a,** Cu NCs **b,** Cu@AlOx$_{n=17}$ and **c,** Cu/AlOx.

The evolution of the copper species as a function of time at -1.1V vs RHE from LCA for the three systems is summarized in **Figure 4g-i**. **Figure S23** reports the original data. The evolution of the XANES spectra as function of time when -1.1 V vs RHE is maintained, clearly shows that the as-synthesized Cu NCs maintained their metallic character, as expected, while Cu@AlOx$_{n=17}$ maintained its mixed oxide-metal composition based on the white line feature. and the LCA fitting (Fig. 4) Interestingly Cu/AlOx shows progressive reduction towards metallic copper corroborating the instability of the Cu|AlOx interface.

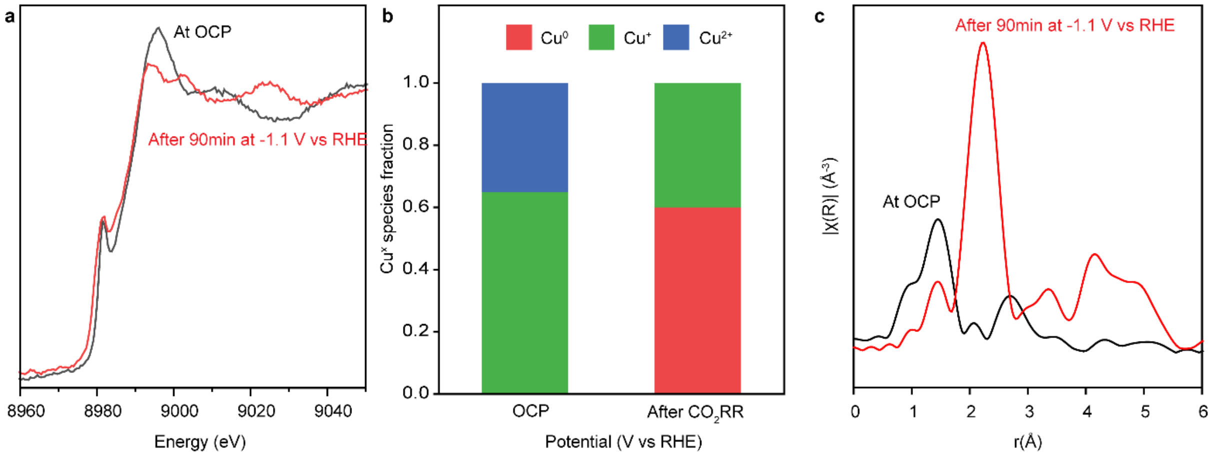

Figure S24. Ex-situ XAS data of Cu@AlOx$_{n=8}$ (partial encapsulation) before and after 90 min of CO_2RR at -1.1 V vs RHE. **a,** XANES spectra. **b,** Cu^{x} species fraction from LCA. **c,** EXAFS spectra.

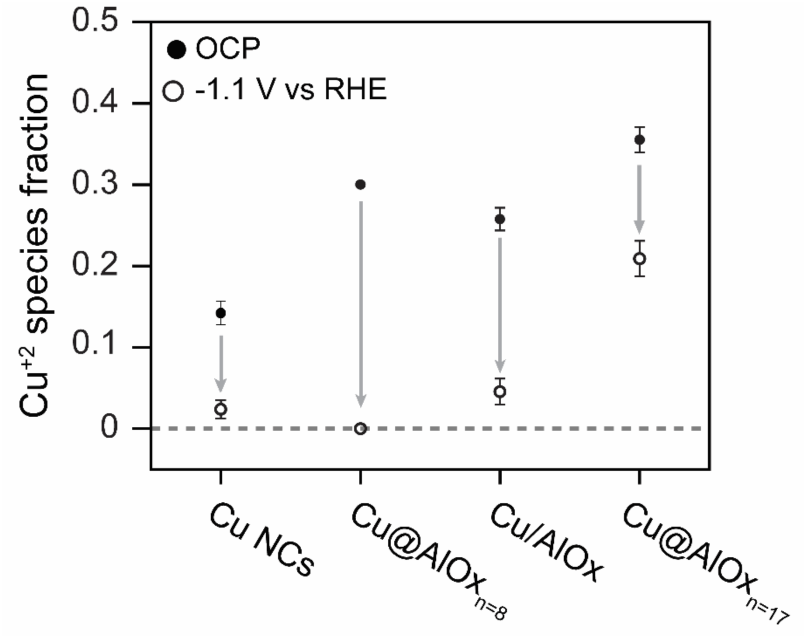

Figure S25. Evolution of Cu^{+2} fraction from OCP to -1.1 V vs RHE for different catalysts based on LCA performed on the XANES data. The Cu^{2+} fraction drops to 0% for all samples, except Cu@AlOx$_{n=17}$. The data are the average of copper fractions extracted from six consecutive XANES experiments and the error bars are the calculated standard deviation.

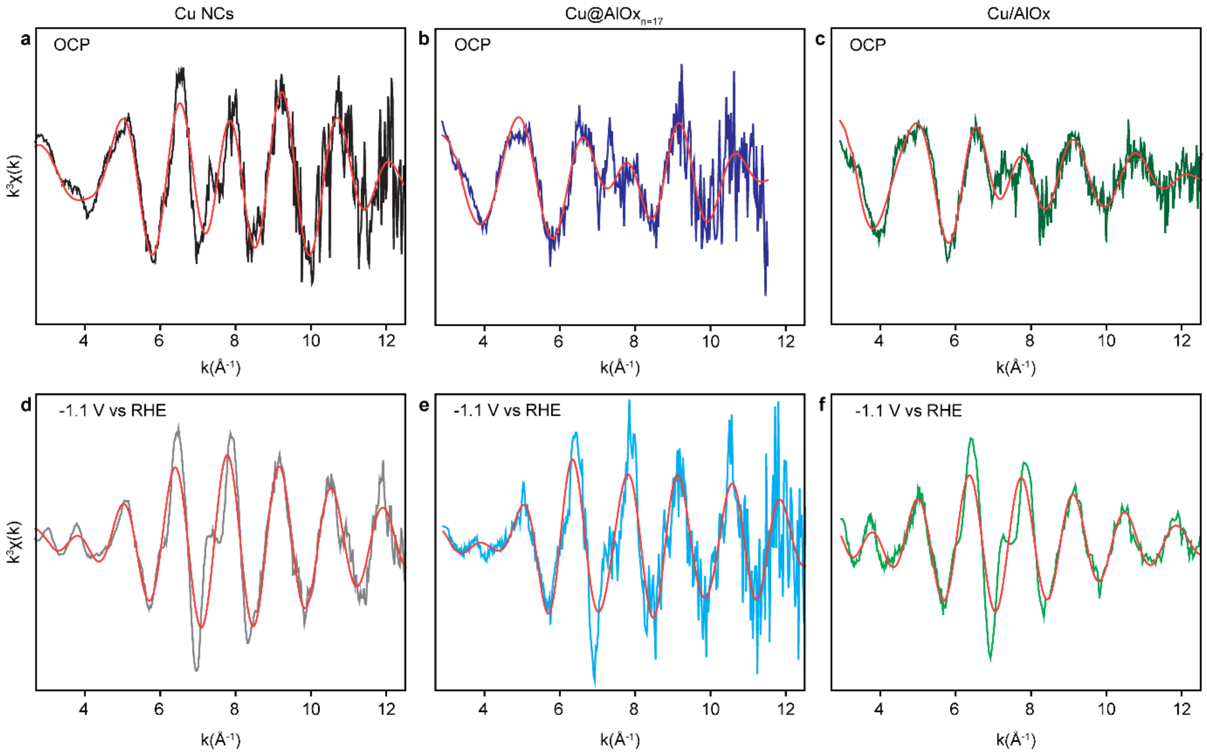

Figure S26. k^3-weighted EXAFS spectra at OCP (dark color) and at -1.1 V vs RHE (light color) of **a, d** Cu NCs (black), **b,e** Cu@AlOxn=17 and **c,f** Cu/AlOx (green) with their respective fittings (red).

In reporting of the EXAFS analysis, in all cases the fitted data range is $3 \leq k$ (Å^{-1}) ≤ 12.5 and the fitting made using a k^3 weighting of the EXAFS data.

The R-factor (R%) is defined as follows as follows:

$$R\% = \Sigma_i^N 1/\sigma_i (\chi_i^e (k) - \chi_i^t (k))^2 \text{ x } 100\%$$

Where χ_i^e and χ_i^t are the experimental and theoretical EXAFS respectively and k is the photo-electron wave-vector (Å^{-1}). σ_i is the uncertainty in the data, with $1/\sigma_i = k_i^n/\Sigma j^N k_i^n (\chi_i^e (k_j))^2$.

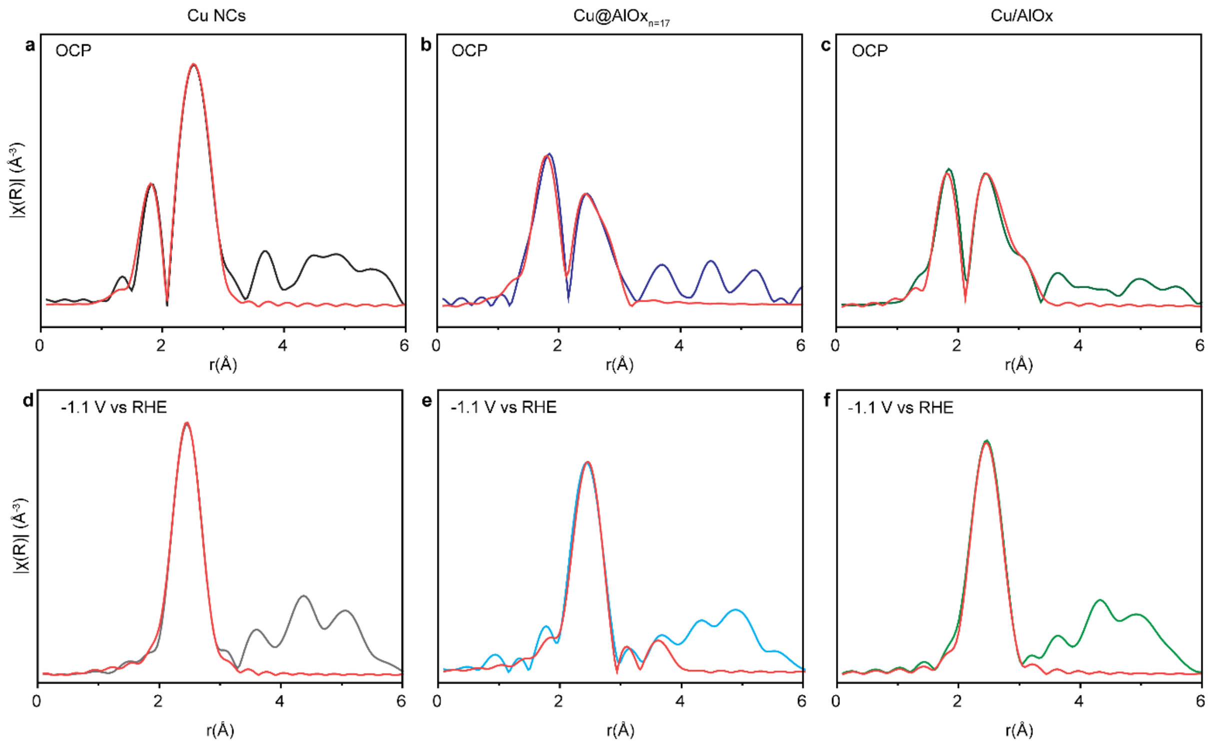

Figure S27. Phase-corrected FT(k^3)-EXAFS spectra at OCP (dark color) and at -1.1 V vs RHE (light color) of **a, d** Cu NCs (black), **b, e** Cu@AlOxn=17, and **c, f** Cu/AlOx (green) with their respective fittings (red).

Figure S27 reports the original FT-EXAFS along with their fitting. Below we write more detailed comments on the data to complement the summary provided in the discussion of Fig. 4 in the manuscript.

At OCP, the EXAFS spectra of the three systems display two pronounced features, one at 1.8 Å and one at 2.53 Å, which are characteristic of Cu-Cu and Cu-O bonds, respectively. The Cu-O bond lengths are more indicative of Cu_2O (1.85 Å) than CuO (1.95 Å), which is in line with LCA from XANES where the fraction of Cu^+ is higher than Cu^{2+} at OCP.

, Upon the application of the cathodic potential, the EXAFS spectra undergo severe changes and become dominated by the Cu-Cu scattering interaction at 2.53 Å, and the appearance of higher shell structure ((r > 3Å) indicative of the presence of an extended metallic copper phase, which is also consistent with the XANES data and analysis. Again, Cu@AlOx$_{n=17}$ behaves differently in that two non-fcc scattering peaks corresponding to Cu-O are preserved in its EXAFS spectrum at -1.1V vs RHE. A shell of O at 1.968 Å can be fitted and correspond to CuO, which is in agreement with the XANES indicating the Cu^{2+} existence at cathodic potential. Instead, the second non-fcc scattering feature at 3.1 Å does not correspond precisely to any of the bulk reference compounds. The fitted Cu-O distance falls somewhere

in between the expected region for CuO and $CuAl_2O_4$ and might reflect an interaction between oxidized copper surface with the alumina matrix, thus we indicate it as Cu-O(Al).

The changes in the scattering features discussed above are accompanied by changes in coordination numbers (N1), bond distances (d) and the Debye-Waller (DW) factor extracted from the EXAFS (Table S4). The changes are dominated by the reduction of the oxidized components initially present and their replacement by metallic fcc copper, as very clearly seen in the Fourier transforms of the k^3-weighted EXAFS data shown in Figure S26.

N1 does have a sensitivity to particle size (specifically for average atomicity of < ca. 1000 atoms).[28,29] Thus, it would be tempting to associate some of the changes in N1 to changes in the average copper particle sizes (smaller sizes -> more surface and defects -> higher N1). However, the errors intrinsic to the determination of the reported values (± 10-20%), derived in large part from the high correlation that exists between the N1 and the DW factor, prevents to make any further correlation with any reliability.

Table S4. Structural data extracted from analysis of Cu K-edge EXAFS at OCP and operando at -1. 1V vs RHE.

**Sample**	**N1 (Cu)**	**d (Å)**	**DWF (Å^2)**
Cu NCs – OCP	3.7	2.51	0.003
Cu NCs – -1.1V vs RHE	11	2.53	0.014
Cu@AlOx$_{n=17}$ – OCP	2	2.50	0.009
Cu@AlOx$_{n=17}$ – -1.1V vs RHE	9.4	2.523	0.014
Cu/AlOx – OCP	1.7	2.51	0.012
Cu/AlOx – -1.1V vs RHE	10.6	2.54	0.015

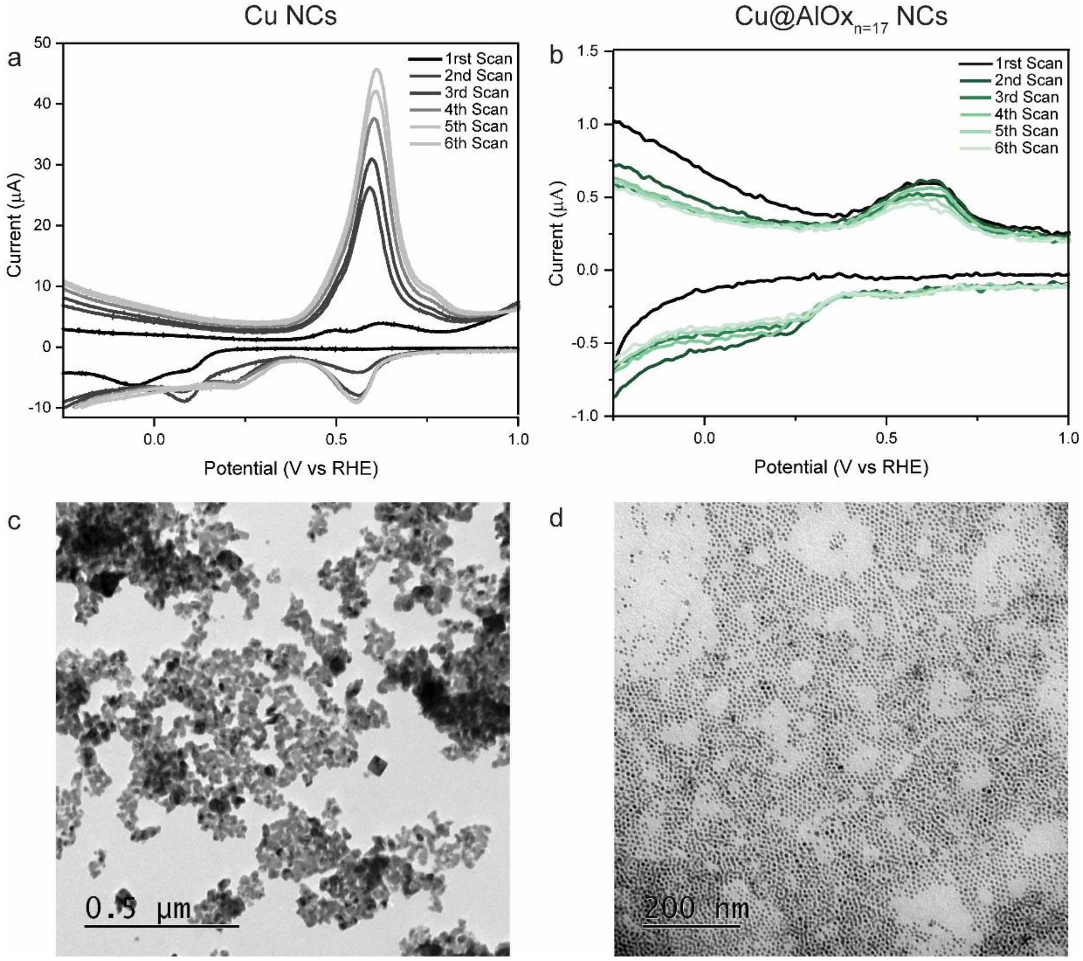

Figure S28. Stability of the Cu@AlOx$_{n=17}$ NCs against surface oxidation/reduction cycles. a,b Successive cycles of SWV for the as-synthesized Cu NCs (**a**) and for the Cu@AlOx$_{n=17}$ (**b**). **c, d** Representative TEM images acquired after the SWV cycles for Cu NCs (**c**) and Cu@AlOx$_{n=17}$ NCs (**d**).

Successive cycles of square wave voltammetry (SWV) were performed on the as-synthesized Cu and Cu@AlOx NCs (**Figure S15a,b**) to assess their resistance against redox cycles. Remarkably, the SWV curves of the encapsulated catalyst do not show change upon cycling. This result is significant because these redox phenomena are one of the triggering events for the reconstruction of the Cu catalysts during start-up and shut-down of the electrochemical cell.[30] Additional TEM images taken for both system after SWV confirm the robustness of the coating and absence of changes for Cu@AlOx$_{n=17}$ (**Figure S15c,d**).

Additional information can be gained from the comparison of the first scans between the two samples. Both samples show the two nearby peaks at around 0.5 V vs RHE on the anodic wave, which correspond to the reduction from Cu^+ to Cu^0. However, only the Cu NCs display a reductive peak at around 0 V vs RHE. This reductive peak was previously ascribed to

surface ligands electrodesorption.[2] The absence of this peak, or any other new feature, in Cu@$AlOx_{n=17}$ NCs indicates that neither the alumina shell with its embedded ligands desorb from the surface nor does the aluminum alloys with copper. Interestingly, the feature corresponding to Cu+ to Cu0 reduction increases upon cycling in the SWV of Cu NCs, which we correlate to the reconstruction pointing at formation of cube-like structure upon oxidation.[31,32]

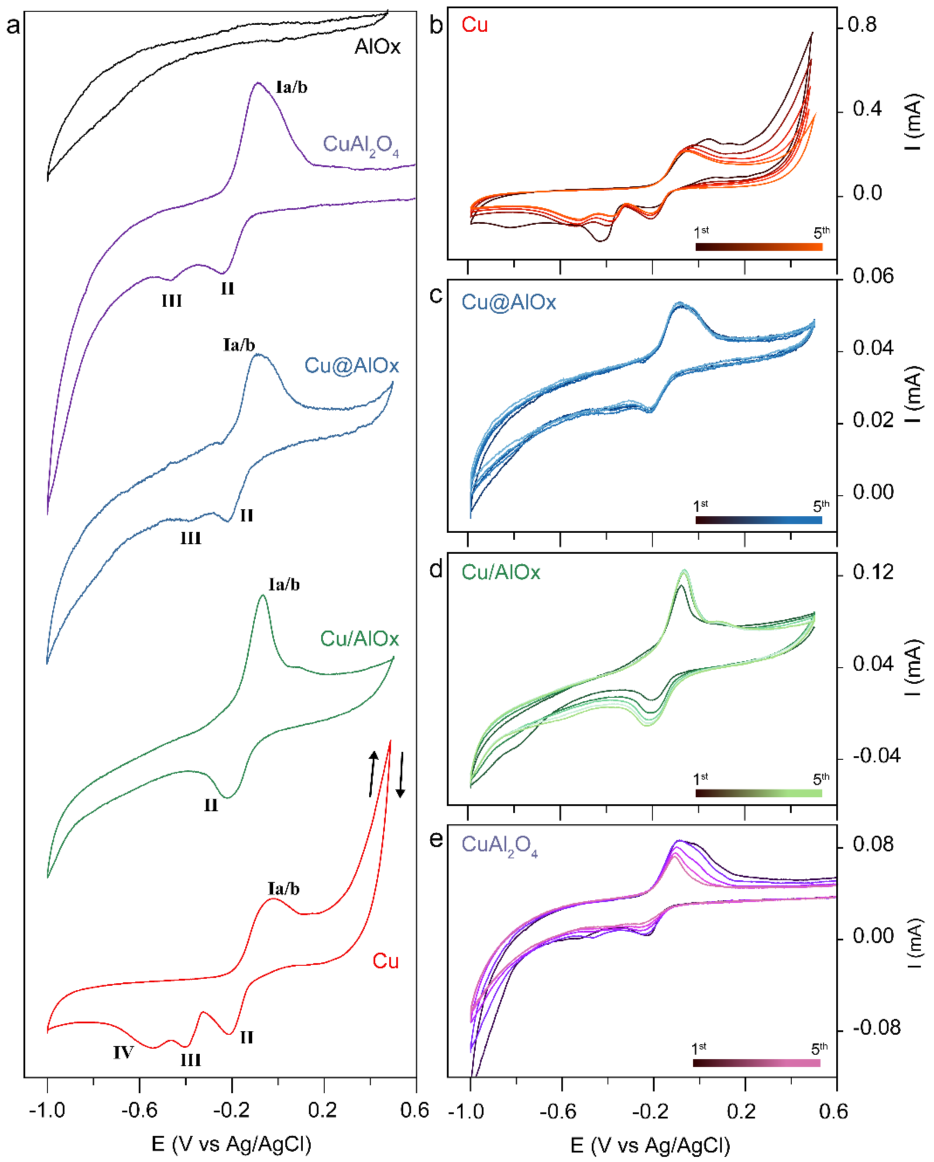

Figure S29. a. Cyclic voltammograms from cathodic to anodic wave, run in 0.1M KHCO3 at 25mV/s for the as-synthesize Cu NCs (red), Cu/AlOx (green), Cu@AlOx (blue), $Cu_2Al_2O_4$ (violet) and AlOx (black). **b-e.** Successive cycle of CVs for Cu NCs (red, **a**), Cu/AlOx (green, **b**), Cu@AlOx (blue, **c**), Cu2Al2O4 (violet, **d**) and AlOx (black, **e**).

Cyclic voltammograms (CV) were acquired on as-synthesized Cu, Cu@AlOx and Cu/AlOx NCs together with commercially available $CuAl_2O_4$ and AlOx synthesized by reactiong TMA with IPA (Fig S29). From literature four different regions could be identified in the CV (Fig S29a).[33–36] First, Ia/b around 0 V vs Ag/AgCl has been ascribed to the oxidation of Cu^0 to Cu^{+2}, where different species can coexist (e.g. CuO, $Cu(OH)_2$, $Cu(OH)_4^{2-}$…). Then, II and III has been ascribed to the reduction of Cu^{+2} to Cu^0 and carbonate species respectively. We hypothesize that IV corresponds to more difficult species to reduce, such as dissolved Cu ions coordinated in the electrolyte. The CV of AlOx does not change across the potential window,

confirming its redox stability and inertness toward CO_2. Interestingly, all the Cu-based samples differ mostly in their region Ia/b which correspond to Cu^{+2} species. Thus, successive CV were performed to interrogate the stability of those Cu^{+2} species (Fig S29b-e). Among the different samples, only the CV of Cu@AlOx does not show major changes, which confirms the stability provided upon encapsulation. Interestingly, a decrease in current in the Ia/b region for the as-synthesized Cu NCs occurs, which might indicate a progressive dissolution of the Cu in the electrolyte (indeed, after 5 CV, the Cu deposited on the electrode is severely removed). In the case of $CuAl_2O_4$, the regions Ia/b is rather similar to Cu@AlOx but changes progressively upon cycling. We speculate that this change in the Cu^{+2} regions might correspond to a reconstruction of the material similar to what has been observed when used during CO_2RR. Lastly, Cu/AlOx Ia/b region upon cycling increase in current meaning that more and more Cu site become accessible. This observation is consistent with a dealumination process and loss of the interface, similar to what observed in XAS during the potential ramp.

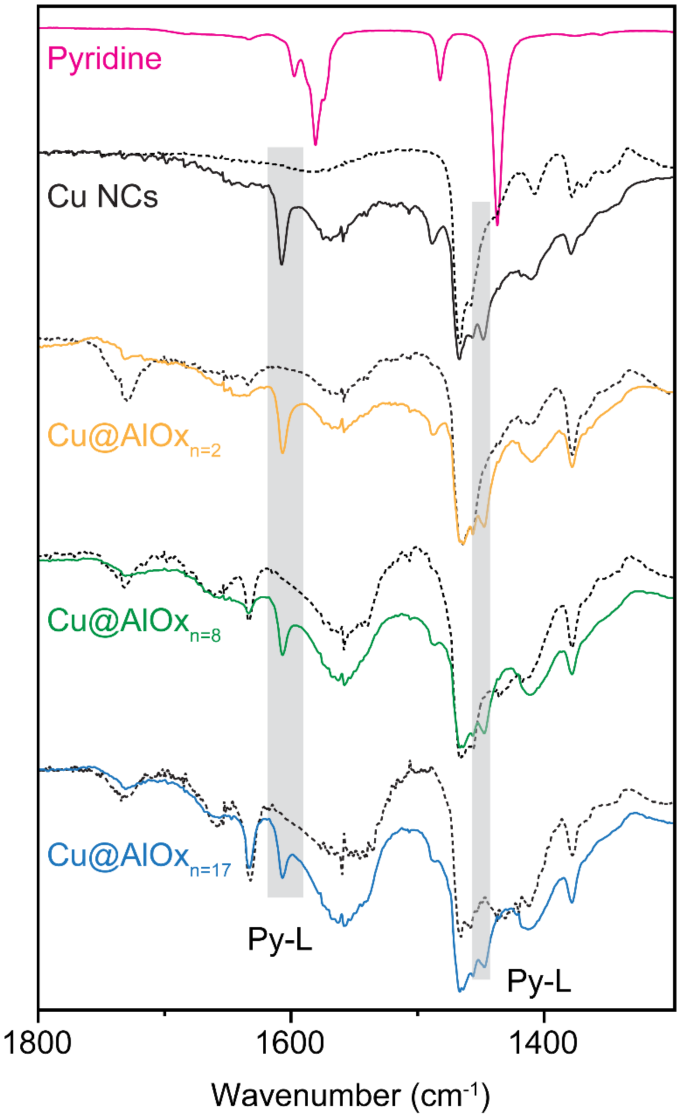

Figure S30. FT-IR spectra of Cu and Cu@AlOx without (dotted line) and with (solid line) contact pyridine for n= 2, 8 and 17 cycles.

Upon contact with pyridine (py), the FT-IR spectra shows the appearance of two new bands at 1607 cm^{-1} and 1445 cm^{-1}, independent of the shell thickness. Those bands have been previously ascribed to the Lewis-bonded pyridine (Py-L) to the Cu surface.[37] The fact that the pyridine reaches the copper surface indicates that the c-ALD-grown alumina shell is a porous network rather than a dense thick coating that buries the copper surface like oxides deposited by gas-phase ALD or carbon coatings.[38,39]

Supplementary Note 2: Use of metal|oxide interface in CO2RR

Examples of Cu|oxide systems for CO_2RR with improved stability have been reported in the literature (see table below). Complete encapsulation of the copper generally results in a catalytic behavior dominated by the coating itself, resulting in either formate or syn-gas production.[40–42] Only when patchy metal|oxide interfaces are engineered, improved Cu performances toward CO2RR are observed.[43,44] Yet rational design of those interfaces is still lacking. Moreover, structural stability is not necessarily achieved due to an unevenly distributed oxide network. As structural change still occurs, establishing clear correlation between the "pre-catalyst" and the observed performances becomes laborious.

Our work establishes a unique synthetic approach to engineer metal| oxide interfaces wherein complete encapsulation improves structural stability while the presence of a porous interface allows the CO_2RR to proceed on copper, thus taking advantage of its unique properties and the accumulated knowledge on this catalytic material. This balance reactivity/stability is achieved thanks to the hybrid organic/inorganic nature of our coating.

Catalyst	Cell-type	Potential	FE of major product	Stability time	Ref
Cu@AlOx	H-cell	-1.1V vs RE	20% CH4	24 h	This work
Cu/SnO2	H-cell	-0.7 V vs RHE	93% CO	1 h	[40]
Cu/In2O3	H-cell	-0.7 V vs RHE	90% syngas	5h	[41]
Cu/SnOx	Flow-cell	-0.55 V vs RHE	90% CO	40 h	[42]
Cu/CeOx	H-cell	-1.2 V vs RHE	55% CH4	12 h	[43]
Cu/BaO	Flow-cell	-0.75 V vs RHE	58% alcohol	20 h	[44]

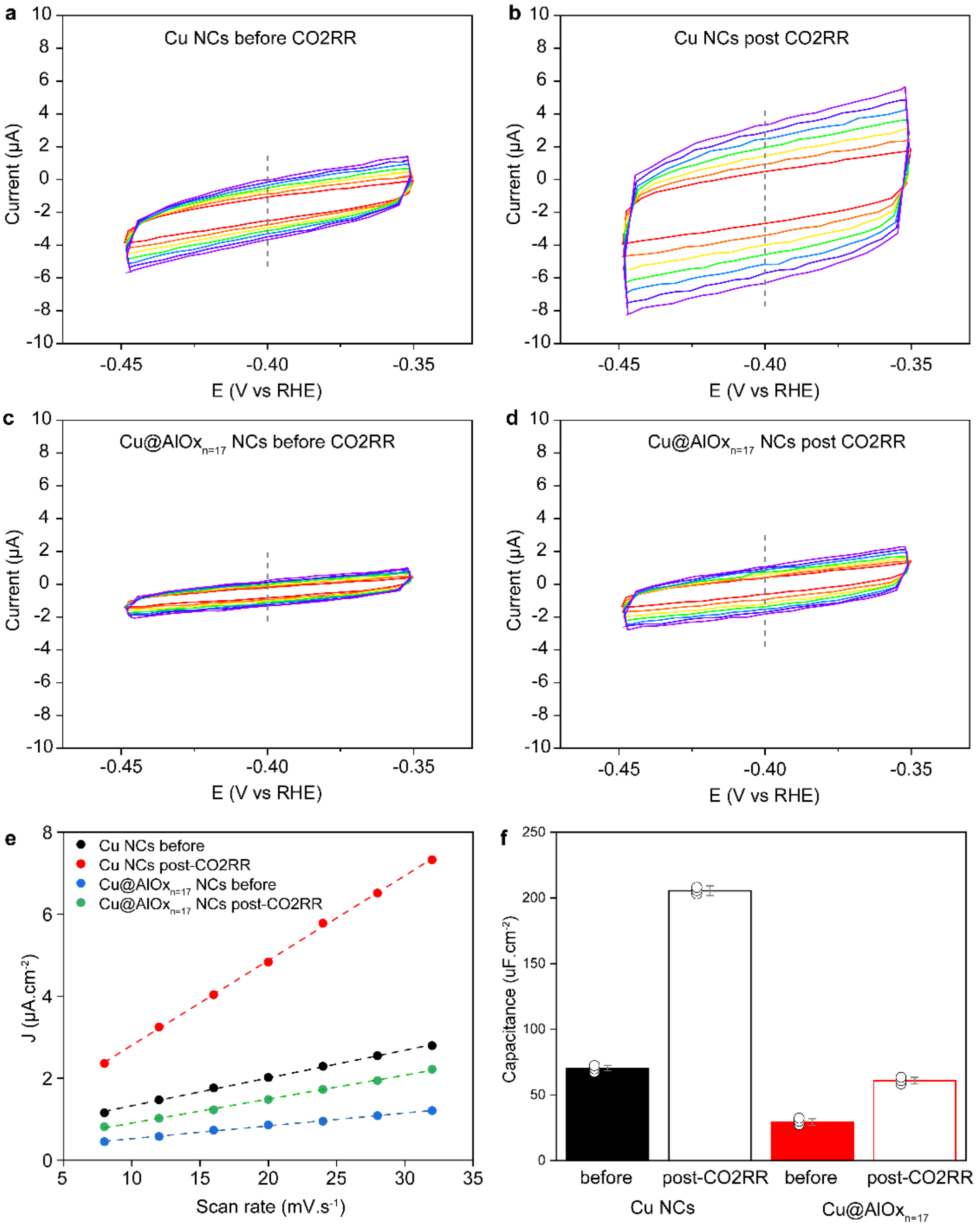

Figure S31. Determination of the electrochemically active surface area (ECSA). a-d. Representative examples of cyclic voltammograms used to determine the capacitance of Cu NCs before (**a**) and post CO_2RR (**b**) and of Cu@AlOx$_{n=17}$ before (**c**) and post CO_2RR (**d**). **e.** Total geometric current density plotted against the scan-rate to extract the sample capacitance. The total geometric current density was calculated from the difference between the charging and discharging current from the cyclic voltammograms taken at 0.4 V vs Ag/AgCl with a geometric surface area of 1.33 cm^2. **f.** Calculated capacitance for Cu NCs and Cu@AlOx$_{n=17}$ NCs before and post CO_2RR. The post CO_2RR capacitance is later used to calculate the ECSA normalized current density by dividing it by the capacitance of the glassy carbon electrode (C_{GC}=30uF/cm^2). The data are the average of three independent experiments

(individual data points are reported in the graph) and the error bars are the calculated standard deviation.

The electrochemically active surface area (ECSA) is key parameter to assess the intrinsic activity of electrocatalysts. Different methodologies exists with all their pros and cons; the most used across the literature are capacitance measurements and metal underpotential deposition UPD (e.g. Pb). The first one requires that the capacitive current only arises from the double layer charging. On the other hand, UPD requires extracting the covering ratio between Cu and the deposited metal to calculate the ECSA. The latter is not trivially accessible in our case because Cu NCs are coated with a porous AlOx shell. Thus, we opted to assess the ECSA by capacitance measurements. In the case of oxide coated, capacitance measurement has been often stated impracticable, mainly two to the insulating properties of the oxide that had an additional capacitive contribution. However, in our case the material remains conductive and the plotted total current density against scan rate leads to a straight line with a regression coefficient above 0.999 in every case, which confirm that the capacitive contribution mainly comes from the double layer charging.[45]

Regarding the evolution of the capacitance for Cu NCs and Cu@AlOx NCs, we note that both increase after CO2RR, although the change is more substantial in Cu NCs. The drastic increase for the Cu NCs is attributed to their reconstruction into scramble catalysts leading to an increase in number of active sites. Instead, we attribute the moderate increase for Cu@AlOx$_{n=17}$ NCs to the reduction of the unpassivated sites on the surface, in line with the evolution of the Cu speciation from the operando XANES.

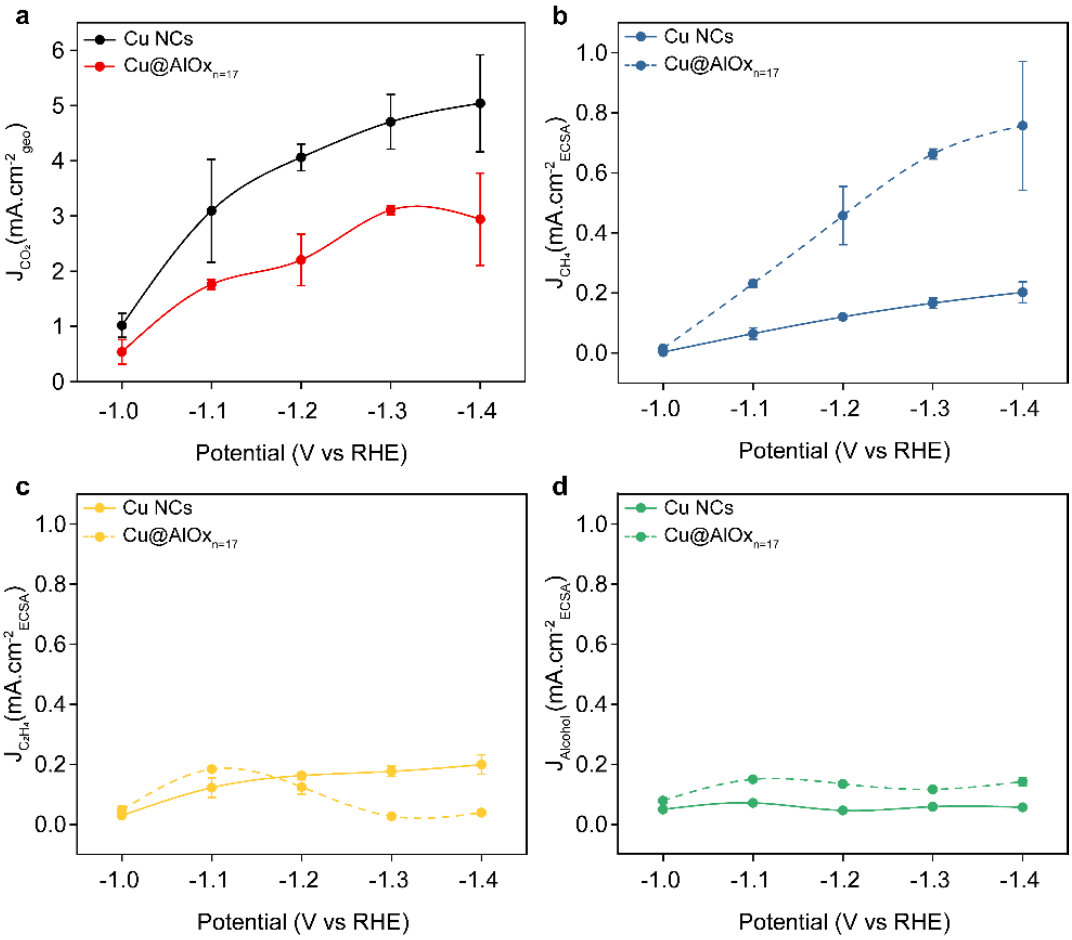

Figure S32. a. Partial current density of CO_2 for Cu NCs and Cu@AlOx$_{n=17}$ normalized by geometric area. **b-d**. ECSA-normalized partial current density of methane (**b**), ethylene (**c**) and alcohol (**d**) for Cu (full line) and Cu@AlOx$_{n=17}$ (dash line). The data are the average of three independent experiments and the error bars are the calculated standard deviation.

The geometric current density for CO2RR drops upon encapsulation , which is in line with the metal oxide shell passivating some active sites on the surface as well as hindering electron transport (Fig S32a). However, the current density normalized by the electrochemical surface area (ECSA) shows the opposite trend with the ECSA-normalized $J_{CO2}$for Cu@AlOx$_{n=17}$ NCs being double compared to the as-synthesized Cu NCs (Fig 5a). This discrepancy indicates that the intrinsic activity of the fewer active sites on Cu@AlOx$_{n=17}$ is higher than those on the as-synthesized Cu NCs.

A more detailed analysis evidences that the partial current density of the methane is clearly enhanced by a factor five in the Cu@AlOx$_{n=17}$ while the ethylene is almost completely suppressed at the most cathodic potentials (Figs S32b,c). Interestingly, the partial current density for other C_{2+} products including ethanol and n-propanol remains unaltered (Fig 5d).

This observation becomes particularly relevant as it indicates that C2+ products are not all suppressed by the shell and alcohols are generated across the entire potential range. We note that methane producing catalysts have been demonstrated to form ethanol in tandem system by favoring CO-CHx.[46] This might provide a hint for future in-depth mechanistic studies. As for now, these data suggest that the ethylene suppression is not correlated to a spatial impediment of C-C- coupling but rather to an electronic effect that suppress one reaction pathway.

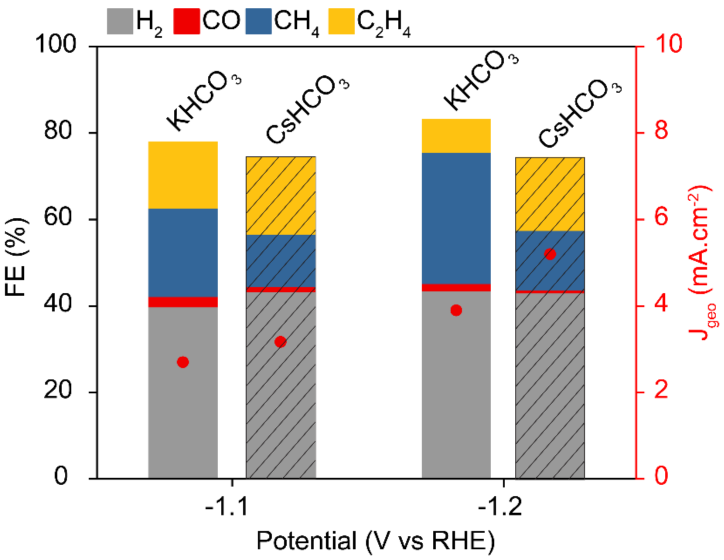

Figure S33. Comparison of the CO_2RR performance of Cu@AlOx$_{n=17}$ NCs in 0.1M $KHCO_3$ and 0.1M $CsHCO_3$. Total FEs for all gaseous products (i.e., H_2, CO, CH_4, C_2H_4) together with the geometric current density (red, right axis) as a function of applied potential. Upon switching the electrolyte from 0.1M $KHCO_3$ and 0.1M $CsHCO_3$ the FE(C_2H_4) is increase at the expanse of FE(CH_4), showing the ability to recover C-C coupling upon manipulation of the microenvironment.

References

1. Hung, L.-I., Tsung, C.-K., Huang, W. & Yang, P. Room-Temperature Formation of Hollow Cu2O Nanoparticles. *Adv. Mater.* **22**, 1910–1914 (2010).

2. Pankhurst, J. R., Iyengar, P., Loiudice, A., Mensi, M. & Buonsanti, R. Metal-ligand bond strength determines the fate of organic ligands on the catalyst surface during the electrochemical CO2reduction reaction. *Chem. Sci.* **11**, 9296–9302 (2020).

3. Loiudice, A., Strach, M., Saris, S., Chernyshov, D. & Buonsanti, R. Universal Oxide Shell Growth Enables in Situ Structural Studies of Perovskite Nanocrystals during the Anion Exchange Reaction. *J. Am. Chem. Soc.* **141**, 8254–8263 (2019).

4. Loiudice, A., Segura Lecina, O., Bornet, A., Luther, J. M. & Buonsanti, R. Ligand Locking on Quantum Dot Surfaces via a Mild Reactive Surface Treatment. *J. Am. Chem. Soc.* **143**, 13418–13427 (2021).

5. Segura Lecina, O. *et al.* Colloidal-ALD-Grown Hybrid Shells Nucleate via a Ligand–Precursor Complex. *J. Am. Chem. Soc.* **144**, 3998–4008 (2022).

6. Coates, J. Interpretation of Infrared Spectra, A Practical Approach. in *Encyclopedia of Analytical Chemistry* (John Wiley & Sons, Ltd, 2006).

7. Dong, C. *et al.* Mechanism studies on thermal dissociation of tri-n-octylamine hydrochloride with FTIR, TG, DSC and quantum chemical methods. *J. Chem. Sci.* **129**, 1431–1440 (2017).

8. Luschtinetz, R., Seifert, G., Jaehne, E. & Adler, H. J. P. Infrared spectra of alkylphosphonic acid bound to aluminium surfaces. *Macromol. Symp.* **254**, 248–253 (2007).

9. Sithole, R. K. *et al.* Synthesis and characterization of Cu3N nanoparticles using pyrrole-2-carbaldpropyliminato Cu(II) complex and Cu(NO3)2 as single-source precursors: The search for an ideal precursor. *New J. Chem.* **42**, 3042–3049 (2018).

10. Köse, D. A. & Necefoğlu, H. Synthesis and characterization of bis(nicotinamide) m-hydroxybenzoate complexes of Co(II), Ni(II), Cu(II) and Zn(II). *J. Therm. Anal. Calorim.* **93**, 509–514 (2008).

11. Biesinger, M. C. Advanced analysis of copper X-ray photoelectron spectra. *Surf.*

Interface Anal. **49**, 1325–1334 (2017).

12. Major, G. H. *et al.* Practical guide for curve fitting in x-ray photoelectron spectroscopy. *J. Vac. Sci. Technol. A* **38**, 061203 (2020).

13. Gharachorlou, A. *et al.* Trimethylaluminum and Oxygen Atomic Layer Deposition on Hydroxyl-Free Cu(111). *ACS Appl. Mater. Interfaces* **7**, 16428–16439 (2015).

14. Liz-Marzán, L. M., Giersig, M. & Mulvaney, P. Synthesis of Nanosized Gold-Silica Core-Shell Particles. *Langmuir* 4329–4335 (1996).

15. Zhang, T. *et al.* Unconventional route to encapsulated ultrasmall gold nanoparticles for high-temperature catalysis. *ACS Nano* **8**, 7297–7304 (2014).

16. Malerba, C. *et al.* Absorption coefficient of bulk and thin film Cu2O | Elsevier Enhanced Reader. *Sol. Energy Mater. Sol. Cells* **95**, 2848–2854 (2011).

17. Calvo-de la Rosa, J., Locquet, A., Bouscaud, D., Berveiller, S. & Citrin, D. S. Optical constants of CuO and ZnO particles in the terahertz frequency range. *Ceram. Int.* **46**, 24110–24119 (2020).

18. Morterra, C., Emanuel, C., Cerrato, G. & Magnacca, G. Infrared study of some surface properties of boehmite (γ-AlO 2H). *J. Chem. Soc. Faraday Trans.* **88**, 339–348 (1992).

19. Shard, A. G. Practical guides for x-ray photoelectron spectroscopy: Quantitative XPS. *J. Vac. Sci. Technol. A* **38**, 041201 (2020).

20. Lizárraga, R., Holmström, E., Parker, S. C. & Arrouvel, C. Structural characterization of amorphous alumina and its polymorphs from first-principles XPS and NMR calculations. *Phys. Rev. B - Condens. Matter Mater. Phys.* **83**, 1–9 (2011).

21. Straumanis, M. E. & Yu, L. S. Lattice parameters, densities, expansion coefficients and perfection of structure of Cu and of Cu–In α phase. *Acta Cryst.* **25**, 676–682 (1969).

22. Sultan, S. *et al.* Interface rich CuO/Al2CuO4 surface for selective ethylene production from electrochemical CO2 conversion. *Energy Environ. Sci.* **15**, 2397–2409 (2022).

23. Wang, X. *et al.* Identifying an Interfacial Stabilizer for Regeneration-Free 300 h Electrochemical CO2Reduction to C2Products. *J. Am. Chem. Soc.* **144**, 22759–22766 (2022).

24. Zhang, T., Yuan, B., Wang, W., He, J. & Xiang, X. Tailoring *H Intermediate Coverage on the CuAl2O4/CuO Catalyst for Enhanced Electrocatalytic CO2 Reduction to Ethanol. *Angew. Chemie - Int. Ed.* (2023) doi:10.1002/anie.202302096.

25. Yin, M. *et al.* Copper oxide nanocrystals. *J. Am. Chem. Soc.* **127**, 9506–9511 (2005).

26. Vavra, J., Shen, T. H., Stoian, D., Tileli, V. & Buonsanti, R. Real-time Monitoring Reveals Dissolution/Redeposition Mechanism in Copper Nanocatalysts during the Initial Stages of the CO2 Reduction Reaction. *Angew. Chemie - Int. Ed.* **60**, 1347–1354 (2021).

27. Yang, Y. *et al.* Operando studies reveal active Cu nanograins for CO2 electroreduction. *Nature* **614**, 262–269 (2023).

28. Benfield, R. E. Mean coordination numbers and the non-metal–metal transition in clusters. *J. Chem. Soc. Faraday Trans.* **88**, 1107–1110 (1992).

29. Jentys, A. Estimation of mean size and shape of small metal particles by EXAFS. *Phys. Chem. Chem. Phys.* **1**, 4059–4063 (1999).

30. Raaijman, S. J., Arulmozhi, N. & Koper, M. T. M. Morphological Stability of Copper Surfaces under Reducing Conditions. *ACS Appl. Mater. Interfaces* **13**, 48730–48744 (2021).

31. Kim, D., Kley, C. S., Li, Y. & Yang, P. Copper nanoparticle ensembles for selective electroreduction of CO2 to C2–C3 products. *Proc. Natl. Acad. Sci. U. S. A.* **114**, 10560–10565 (2017).

32. Li, Y. *et al.* Electrochemically scrambled nanocrystals are catalytically active for CO2-to-multicarbons. *Proc. Natl. Acad. Sci. U. S. A.* **117**, 9194–9201 (2020).

33. Tromans, D. & Sun, R. *Anodic Behavior of Copper in Weakly Alkaline Solutions*. *J. Electrochem. Soc* vol. 139 (1992).

34. González, S., Pérez, M., Barrera, M., González Elipe, A. R. & Souto, R. M. Mechanism of copper passivation in aqueous sodium carbonate-bicarbonate solution derived from combined X-ray photoelectron spectroscopic and electrochemical data. *J. Phys. Chem. B* **102**, 5483–5489 (1998).

35. Giri, S. D. & Sarkar, A. Electrochemical Study of Bulk and Monolayer Copper in

Alkaline Solution. *J. Electrochem. Soc.* **163**, H252–H259 (2016).

36. Wang, L., Gupta, K., Goodall, J. B. M., Darr, J. A. & Holt, K. B. In situ spectroscopic monitoring of CO2 reduction at copper oxide electrode. *Faraday Discuss.* **197**, 517–532 (2017).

37. Busca, G. FT-IR study of the surface of copper oxide. *J. Mol. Catal.* **43**, 225–236 (1987).

38. Bhardwaj, A. A. *et al.* Ultrathin Silicon Oxide Overlayers Enable Selective Oxygen Evolution from Acidic and Unbuffered pH-Neutral Seawater. *ACS Catal.* **11**, 1316–1330 (2021).

39. Yoo, J. M., Shin, H., Chung, D. Y. & Sung, Y. E. Carbon Shell on Active Nanocatalyst for Stable Electrocatalysis. *Acc. Chem. Res.* **55**, 1278–1289 (2022).

40. Li, Q. *et al.* Tuning Sn-Catalysis for Electrochemical Reduction of CO2 to CO via the Core/Shell Cu/SnO2 Structure. *J. Am. Chem. Soc.* **139**, 4290–4293 (2017).

41. Xie, H. *et al.* Boosting Tunable Syngas Formation via Electrochemical CO2 Reduction on Cu/In2O3 Core/Shell Nanoparticles. *ACS Appl. Mater. Interfaces* **10**, 36996–37004 (2018).

42. Ye, K. *et al.* In Situ Reconstruction of a Hierarchical Sn-Cu/SnOx Core/Shell Catalyst for High-Performance CO2 Electroreduction. *Angew. Chemie - Int. Ed.* **59**, 4814–4821 (2020).

43. Varandili, S. B. *et al.* Synthesis of Cu/CeO2-x Nanocrystalline Heterodimers with Interfacial Active Sites to Promote CO2 Electroreduction. *ACS Catal.* **9**, 5035–5046 (2019).

44. Xu, A. *et al.* Copper/alkaline earth metal oxide interfaces for electrochemical CO2-to-alcohol conversion by selective hydrogenation. *Nat. Catal. 2022 512* **5**, 1081–1088 (2022).

45. Trasatti, S. & Petrii, O. A. Real Surface Area Measurements in Electrochemistry. *Pure Appl. Chem.* **63**, 711–734 (1991).

46. Iyengar, P., Kolb, M. J., Pankhurst, J. R., Calle-Vallejo, F. & Buonsanti, R. Elucidating the Facet-Dependent Selectivity for CO2Electroreduction to Ethanol of

Cu-Ag Tandem Catalysts. *ACS Catal.* **11**, 4456–4463 (2021).
